# Preparation and Characterization of ACE2 Receptor Inhibitor-Loaded Chitosan Hydrogels for Nasal Formulation to Reduce the Risk of COVID-19 Viral Infection

**DOI:** 10.1021/acsomega.1c05149

**Published:** 2022-01-14

**Authors:** Barbara Vörös-Horváth, Pavo Živković, Krisztina Bánfai, Judit Bóvári-Biri, Judit Pongrácz, Gábor Bálint, Szilárd Pál, Aleksandar Széchenyi

**Affiliations:** † Institute of Pharmaceutical Technology and Biopharmacy, Faculty of Pharmacy, University of Pecs, Rókus u. 2, 7624 Pécs, Hungary; ‡ Department of Chemistry, Josip Juraj Strossmayer University of Osijek, Ulica Cara Hadrijana 8/A, HR-31000 Osijek, Croatia; § Department of Pharmaceutical Biotechnology, Faculty of Pharmacy, University of Pecs, Rókus u. 2, 7624 Pécs, Hungary

## Abstract

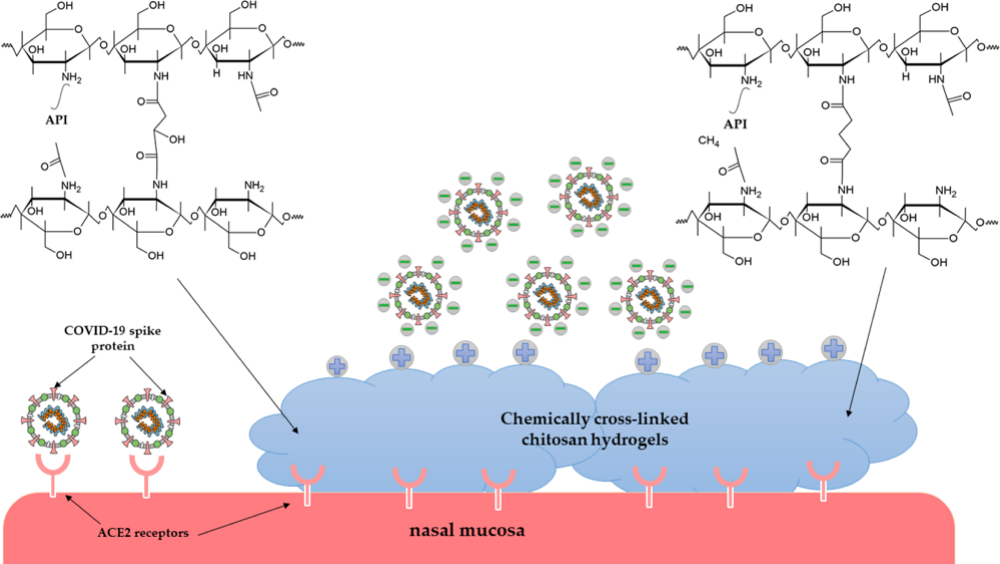

The COVID-19 virus is spread by pulmonary droplets. Its high infectivity is caused by the high-affinity binding of the viral spike protein to the ACE2 receptors on the surface of respiratory epithelial cell membranes. The proper hydration of nasal mucosa plays an essential role in defense of bacterial and viral infections. Therefore, a nasal formulation, which can moisture the nasal mucosa and contains the ACE2 receptor inhibitor, can reduce the risk of COVID-19 infection. This article presents a systematic study of the preparation of chitosan hydrogels with dicarboxylic acids (malic and glutaric acid) and their detailed characterization (Fourier transform infrared spectroscopy, determination of cross-linking efficiency, rheological studies, thermal analysis, and swelling kinetics). The results confirm that chemically cross-linked chitosan hydrogels can be synthesized using malic or glutaric acid without additives or catalysts. The adsorption capacity of hydrogels for three different ACE2 inhibitors, as APIs, has also been investigated. The API content of hydrogels and their mucoadhesive property can provide an excellent basis to use the hydrogels for the development of a nasal formulation in order to reduce the risk of SARS-CoV 2 infection.

## Introduction

In 2019 December, a new type of SARS virus, the COVID-19, appeared in China’s Wuhan Province, which has spread worldwide within a few months, causing a pandemic. Many research groups are investigating the cause of its high infectivity. The accurate knowledge of the virulence factors of this new type of coronavirus is very important^1^ because mainly these determine its reproduction number (R), which is defined as the average number of secondary transmissions from one infected person. If R is greater than 1, the epidemic is growing. The estimated summary R for COVID-19 is 2.87, but it can differ by geographic regions and climates, for example, the highest R was reported to be 6.32 for France in the European region.^2^

The COVID-19 is spread by pulmonary droplets, mainly through the nasal respiratory pathway, where it binds to nasal epithelial cells and starts to replicate.^3^ One of the most important virulence factors is the structure of the viral spike protein, which determines the entry of the virus into the human cells.^1^ The spike protein binds with a high affinity to ACE2 receptors on the surface of respiratory epithelial cell membranes,^4^ which are found in vast numbers on the nasal mucosa, making the virus most easily get through the nasal passage.^5^ Nasal clearance is a mechanism that prevents entry of the contaminants and pathogens into the human body, but it is not effective if the nasal mucosa is not hydrated properly.^6^

The virulence of the COVID-19 could be significantly reduced if these two factors, spike protein binding to ACE2 receptors and hydration of nasal mucosa, could be addressed effectively. Our suggestion for this purpose is to formulate a nasal hydrogel that contains ACE2 receptor inhibitors, which can act locally to prevent the primary virus replication and provide mucosal hydration for adequate nasal clearance.

Several molecules are currently being tested as potential candidates against COVID-19 viral infection, including compounds of the natural origin.^7^ Many of them are proven to inhibit the binding of spike proteins to ACE2 receptors such as baicalin,^8,9^ emodin,^10,11^^,^ and glycyrrhizin acid.^12−14^ The appropriate material for a nasal hydrogel can also prevent the COVID-19 virus from reaching the epithelial cell surface and binding to ACE2 receptors. It has been proven that the COVID-19 has a negative surface potential;^15^ thus, a hydrogel with a positive surface potential can bind the virus with electrostatic interactions.

Chitosan is a natural polysaccharide that has been increasingly used in pharmaceutical research applications as it is non-toxic, biocompatible, and biodegradable.^16^ It is also used to develop hydrogels with mucoadhesive properties.^17^ Chitosan consists mainly of glucosamine and N-acetylglucosamine. It has a positive surface charge under physiological conditions due to the protonation of amino groups. Therefore, the virus can adhere to the chitosan-based hydrogel covering the nasal mucosa and then be excreted with the nasal secretions.

The chitosan-based hydrogels can be physically or chemically cross-linked.^18,19^ The chemical cross-linked ones are mainly formed by covalent bonds, with the reaction between the amino groups of chitosan and reactive functional groups, such as carbonyl or carboxyl groups.^20^ They are robust and less rapidly degradable networks; that is why they are resistant to the physiological environment. However, their disadvantages are that the residue of cross-linking agents (e.g., formaldehyde, glutaraldehyde, and epoxy derivatives) can irritate the nasal mucosa, which is why we suggest applying non-toxic cross-linking agents that can be used without additional catalyst or solvent for cross-linking. When using chitosan hydrogels for nasal administration, one of their main properties is their viscosity, which should not exceed 500 mPa·s.^17^

This study aimed to prepare chemically cross-linked chitosan hydrogels with non-toxic dicarboxylic acids via the reaction of their carboxyl groups with the free amino groups of chitosan. Malic acid and glutaric acid have been chosen for the experiments because chitosan can be dissolved in their aqueous solution; therefore, there is no need for an additional solubilizing agent. Because these dicarboxylic acids are non-toxic, the biocompatibility of hydrogels is ensured. A study was performed, where the reaction time and temperature were systematically changed, and the properties of the resulting hydrogels were investigated. For the final formulation, three natural compounds have been used: emodin, baicalin, and glycyrrhizic acid, which can inhibit the interaction of the spike protein of COVID-19 with ACE2 receptors in the nasal mucosa.

## Results and Discussion

### Infrared Spectroscopy of Hydrogels

**Figure 1:**
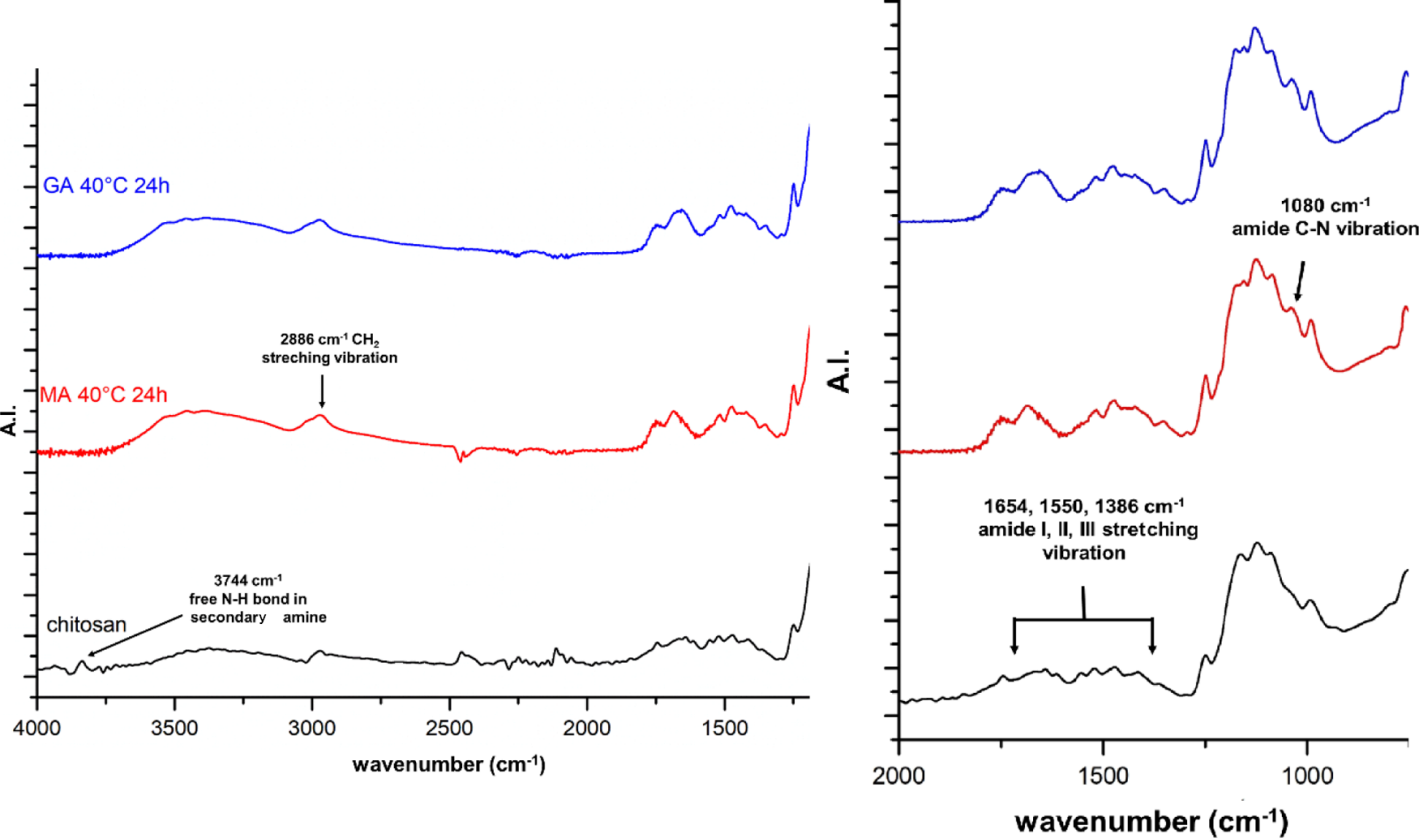
FTIR spectra of chitosan and two different hydrogels.

Figure 1 shows the Fourier transform infrared (FTIR) spectra of chitosan and two different hydrogels. In the spectra of applied chitosan, all the characteristic peaks were found, reported by other research groups.^21,22^ The peak at 903 cm^–1^ is characteristic of the stretching vibration of C–O–C bonds in the glucosamine ring and the saccharide ring’s out-of-plane bending vibration. The stretching vibration of C–O–C bonds in the glucosamine ring can be observed at 1031 cm^–1^. The peak at 1071 cm^–1^ belongs to CH_2_–CO bonds in the chitosan monomer. The chitosan is not fully deacetylated, therefore it contains acetyl groups, and its characteristic peaks can also be observed in the spectra. The amide I peak at 1654 cm^–1^ is attributed to the out-of-plane bending vibration of the C=O group. The sharp, low-intensity peak at 1550 cm^–1^ is attributed to the amide II peak, where the in-plane bending vibration of N–H and the stretching vibration of the C–N bond overlapped. The amide III peak at 1386 cm^–1^ is attributed to the overlapping of the in-plane-scissoring vibration of the N–H bond and the stretching vibration of the C–N bond. At a 2886 cm^–1^ wavenumber, the symmetric and asymmetric CH_2_ stretching vibrations can be seen, attributed to the stretching vibration of the C–H bond in the pyranose ring. The wide, medium intensity peak at 3292 cm^–1^ belongs to the symmetric stretching vibration of the −NH_2_ functional group. In the chitosan spectra, a sharp, low-intensity peak can be seen at 3744 cm^–1^, which is a characteristic out-of-plane vibration frequency peak of the free N–H bond of secondary amines.

In the FTIR spectra of different hydrogels, the characteristic peaks of the polysaccharide structure of chitosan and the amide peaks can also be observed, as described for the pure chitosan above. While comparing the spectra of chitosan and hydrogels, three differences have been noticed. In the case of hydrogels, the characteristic wide, low-intensity peak of −N–H stretching vibration was shifted from 3292 to 3371 cm^–1^ wavenumber, and its intensity increased because of the overlapping of O–H with N–H stretching vibrations and interhydrogen bonds of the polysaccharide chains. In the spectra of hydrogels, the out-of-plane vibration frequency of the free N–H bond disappeared; furthermore, a new peak can be observed at 1081 cm^–1^, which is the characteristic peak of C–N stretching vibration in secondary amides.

We aimed to prove that a chemical cross-linked hydrogel can be prepared only with dicarboxylic acids, such as malic and glutaric acid. However, because of the presence of acetamide groups of chitosan, it is challenging to identify and distinguish the amide bonding, which is formed through the successful cross-linking reaction of chitosan chains. Therefore, instead of the amide I, II, and III peaks, the characteristic peak of C–N vibration was chosen at 1080 cm^–1^, which cannot be observed in the pure chitosan spectra. Following the progress of the cross-linking reaction, the intensity of this peak was compared with the intensity of CH_2_ vibration peak at 2886 cm^–1^, as it can be considered as the inner standard.^23^

Using malic acid, the amide bond formed after 3 h at 298.15 and 313.15 K, but the cross-linking at 323.15 K reaction temperature already formed after 1.5 h. Similarly, in the case of glutaric acid, the amide linkage can be observed after a 3 h reaction time at 298.15 K, but 1.5 h is enough for the bond formation at 313.15 and 323.15 K. Following the cross-linking reaction, it can be observed that the ratio of intensity between the peaks of C–N and CH_2_ amide groups increases if the reaction time increases (Table 1).

**Table 1:** Influence of Reaction Time on Amide Bond Formation of Hydrogels^a^

malic acid				glutaric acid			
sample	*T*_reaction_ (K)	*t*_reaction_ (h)	int. ratio_CN/CH_2__	sample	*T*_reaction_ (°C)	*t*_reaction_ (h)	int. Ratio_CN–CH_2__
chitosan			0.42	chitosan			0.42
MA 251.5	298.15	1.5	0.75	GA 251.5	298.15	1.5	0.85
MA 253		3	2.07	GA 253		3	1.93
MA 254.5		4.5	2.09	GA 254.5		4.5	2.24
MA 256		6	2.26	GA 256		6	2.35
MA 258		8	2.30	GA 258		8	2.80
MA 2524		24	2.92	GA 2524		24	3.06
MA 401.5	313.15	1.5	0.94	GA 401.5	313.15	1.5	1.70
MA 403		3	2.00	GA 403		3	1.86
MA 404.5		4.5	2.01	GA 404.5		4.5	2.18
MA 406		6	2.05	GA 406		6	2.27
MA 408		8	2.42	GA 408		8	3.12
MA 4024		24	2.87	GA 4024		24	3.22
MA 501.5	323.15	1.5	2.11	GA 501.5	323.15	1.5	1.84
MA 503		3	2.62	GA 503		3	2.17
MA 504.5		4.5	2.69	GA 504.5		4.5	2.25
MA 506		6	2.88	GA 506		6	2.32
MA 508		8	3.01	GA 508		8	3.17
MA 5024		24	3.13	GA 5024		24	3.46

a For calculating the absorbance intensity ratio, the intensities of characteristic infrared frequencies of stretching vibration of C–N in the amide bond (1081 cm^–1^) and stretching vibration of C–H in the pyranose ring (2884 cm^–1^) have been used.

### Determination of Cross-Linking Degree

**Figure 2:**
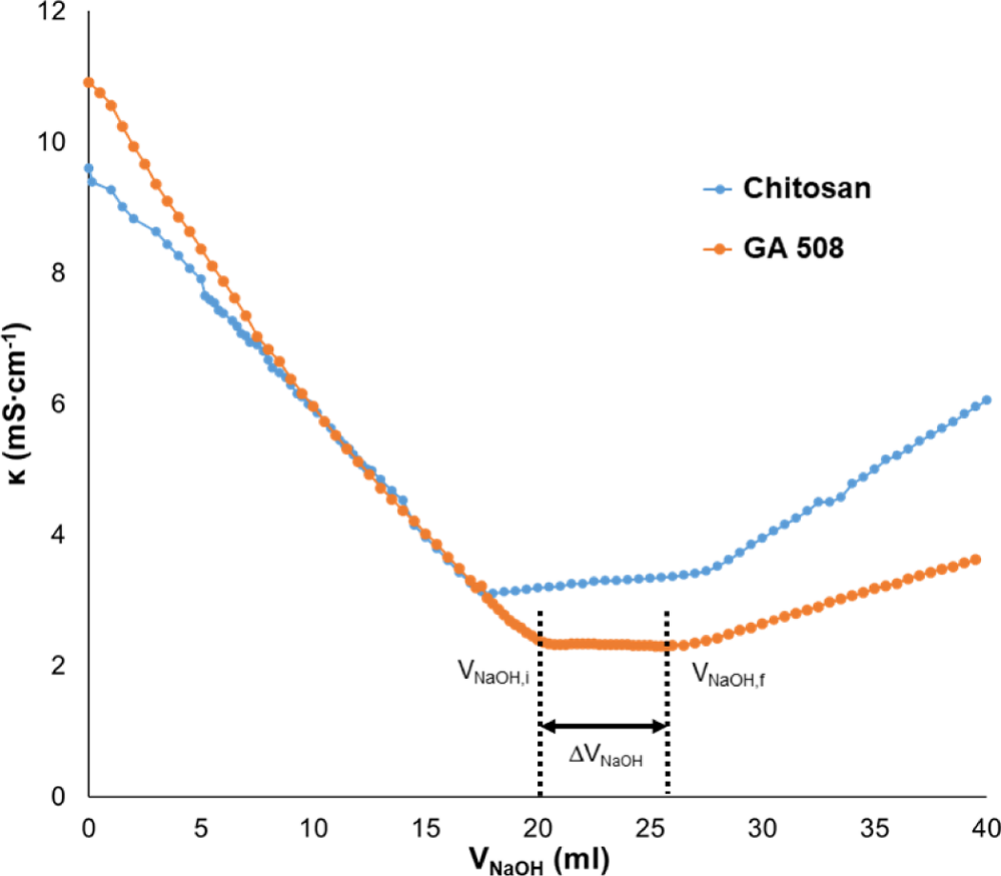
Conductometric titration curve for chitosan and the GA C508 chitosan hydrogel.

An acidic solution of chitosan and different hydrogels were titrated with NaOH solutions, and their conductivity was measured. In Figure 2, conductivity versus the added volume of NaOH is presented; three different regions can be differentiated on the curves. In the first region of the curve, the conductivity of samples decreased if NaOH was added. It is caused by the neutralization of H^+^ of HCl in the solutions. The second region belongs to the neutralization of protonated amino groups in chitosan. The curve’s third region is associated with the addition of Na^+^ and OH^–^ ions to the solution. The volume intercept between the *V*_NaOH_i__ and *V*_NaOH_f__ gives the volume in which the amino groups are neutralized (Δ*V*_NaOH_), with this value, the number of moles of amino groups can be calculated.

The results show that the number of free amino groups decreased with increasing reaction temperature and time. The results confirmed the successful cross-linking reaction of hydrogels. The relative cross-linking (CR) degree can be calculated from the difference of free amino group number between chitosan (*n*_a_^chit^) and hydrogels (*n*_a_^hg^)
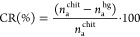


The cross-linking efficacy (CE) can be calculated with the following formula
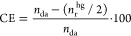
where *n*_r_^chit^ is the mole number of the reacted amino group in chitosan hydrogels and *n*_da_ is the mole number of dicarboxylic acid in the reaction medium.

The CR and CE values increase with the increase of the reaction time and temperature. Comparing the results at the same reaction temperature and time, it can be seen that higher CR values can be achieved by using glutaric acid as a cross-linking agent: for example, the CR values are 30.95 and 52.38% in the case of MA 503 and GA 503 samples, respectively. On the other hand, the CE is lower than 11% in every case because of the excess use of dicarboxylic acids. The number of free amino groups, CR, and CE values of every sample can be found in Supporting Information material, Table S1.

### Determination of Flow Properties

**Figure 3:**
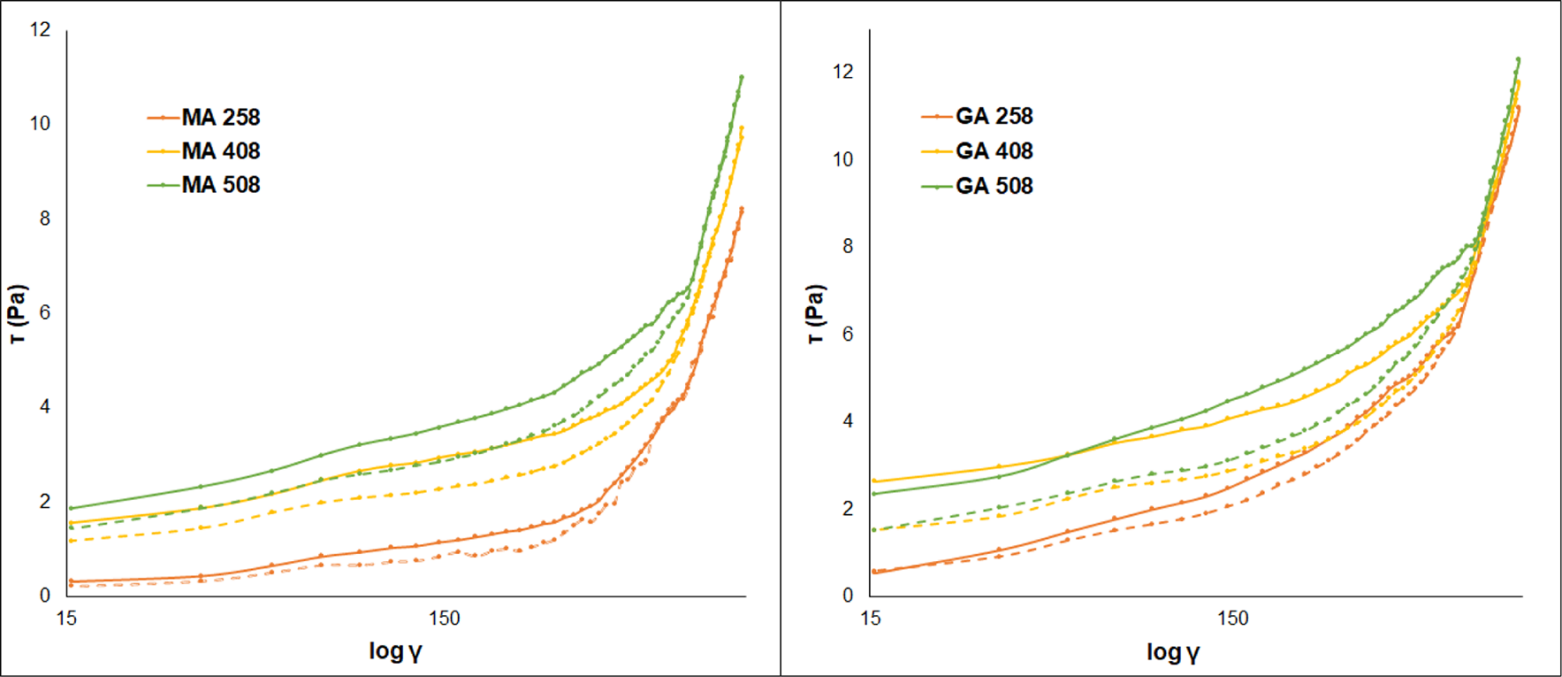
Flow curves of chitosan hydrogels cross-linked with malic and glutaric acid at different reaction temperatures. The continuous lines represent the upward curves, and the dotted lines represent the downward curves.

The flow curves of chitosan hydrogels cross-linked with malic acid and glutaric acid are shown in Figure 3. The shape of flow curves shows Bingham plasticity and a thixotropic behavior, characteristic of an organized network structure in a gel system. The rheological parameters were calculated using the Bingham plasticity mathematical model.

The yield stress values (τ_B_) and the Bingham viscosity (η_B_) increase with increasing reaction temperature and time, both for glutaric and malic acid. If the hydrogel has a higher degree of cross-linking, increasing its viscosity, higher shear stress is needed for its structure destruction. For example, τ_B_ is 0.018 Pa in the case of the MA 251.5 sample, and this value is calculated at a higher reaction temperature; for the MA 501.5 sample, τ_B_ is 0.941 Pa. The τ_B_ and η_B_ values are higher for glutaric acid cross-linked hydrogels than for malic acid cross-linked ones, at each reaction temperature and time point. The GA 5024 sample has the highest τ_B_ and η_B_ values, 2.390 Pa and 9.12 × 10^–3^ Pa·s, respectively.

**Figure 4:**
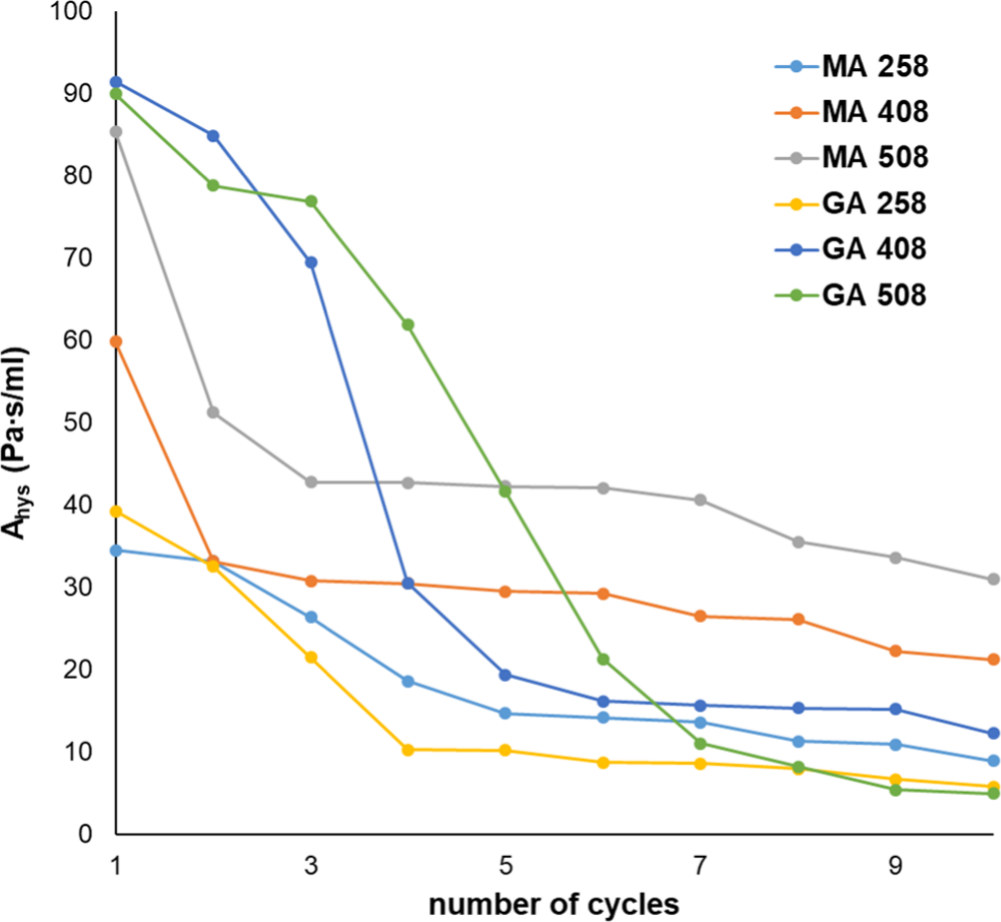
Evaluation of thixotropic behavior of different hydrogels using the hysteresis area method.

The value of the hysteresis area (*A*_hys_) is an indicator of the degree of gel system destructuration; higher values for the thixotropic area indicate higher thixotropy. The results show that the thixotropic behavior will be more pronounced with increasing reaction temperature, indicated by the increase of the hysteresis area. All gels recoup their structural integrity after the applied shear stress is withdrawn, which is attributed to the attainment of an organized gel structure. For the evaluation of thixotropic behavior using the hysteresis area method, the gel samples were sheared with increasing the shear rate (upward curve) immediately followed by decreasing the shear rate (downward curve). After each cycle, the decrement in the hysteresis area can be observed for all samples (Figure 4). The upward and downward curves are almost completely overlapped after the 10th cycle, but all hydrogels retain their Bingham plastic behavior after about 24 h of relaxation time. The flow curves can be seen in Supporting Information material Figure S4. As we described above, the hysteresis area increases with reaction time and temperature, which is caused by the higher cross-linking density of hydrogels. The hysteresis area relates to the structural breakdown of the hydrogels: the area is higher if the hydrogel is densely cross-linked because, in this case, more links are broken due to increasing shear stress. The rheological examinations confirmed the result of the cross-linking determination that the glutaric acid cross-linked hydrogels are denser and have a firmer structure because of their higher hysteresis area.

The dynamic viscosity of gels also increased with increasing reaction time and temperature; besides, using glutaric acid as cross-linking agent resulted in a more viscous gel than using malic acid. The increase in viscosities can be due to forming a more robust structure through the chemical bond between chitosan amino groups and the carboxyl groups.

**Figure 5:**
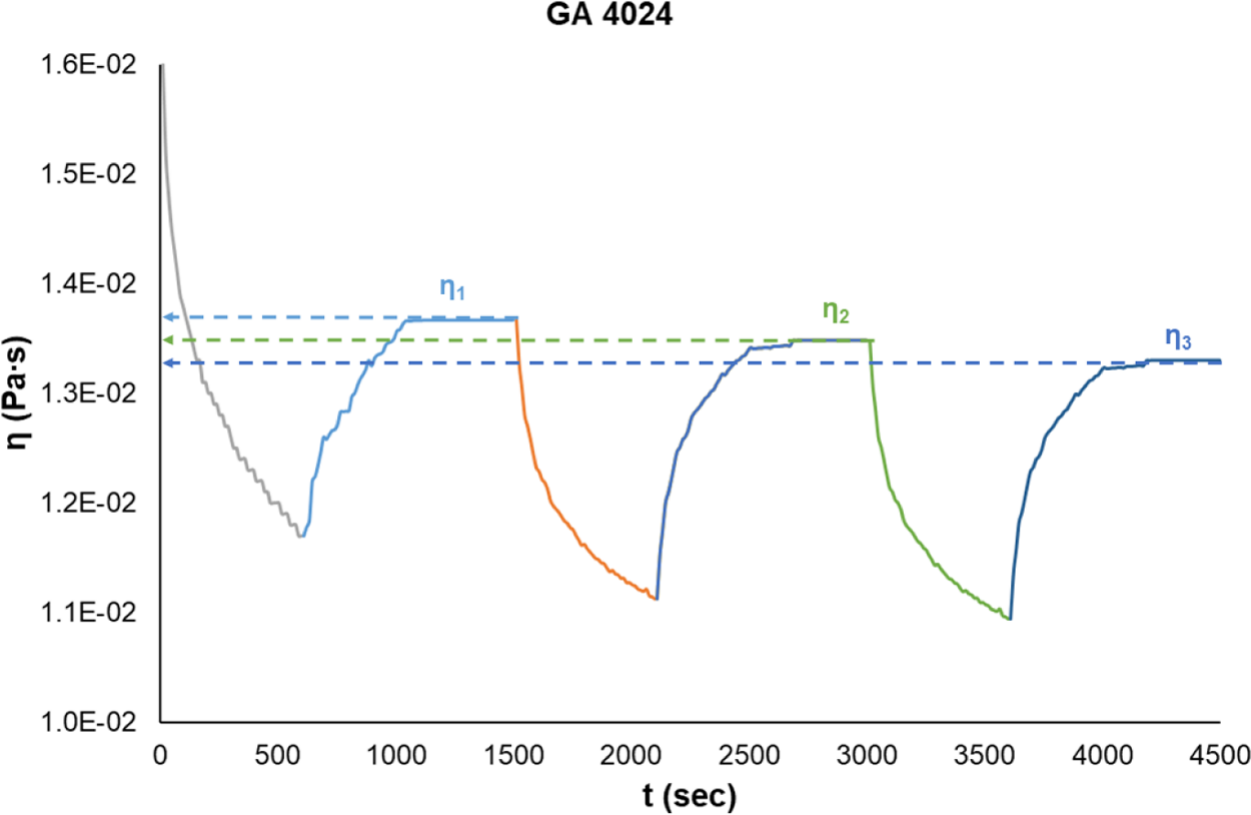
Time-dependent viscosity of the GA 4024 chitosan hydrogel.

One of the specific properties of Bingham plastic fluids is their time-dependent rheological behavior. All hydrogels showed time-dependent viscosity, and the hydrogels are viscoelastic. In Figure 5, the results of the time-dependent viscosity experiment of the GA 4024 sample can be seen. The initial viscosity is 1.650 × 10^–2^ Pa·s, and after 10 min of shearing, the viscosity decreases to 1.17 × 10^–2^ Pa·s. In the relaxation interval (20 min), the hydrogel regenerates and recoups its more viscous state, but after the relaxation, the viscosity value is only 85.42% of the initial value, 1.37 × 10^–2^ Pa·s. The viscosity decreases after each interval; after the second one, its value is 84.28%, and after the third, it is only 68.40%. The velocity of regeneration can be determined from the time-dependent regeneration, whose values were 9.84 × 10^–5^, 1.18 × 10^–4^, and 1.18 × 10^–4^ Pa·s/min, in the first, second, and third interval, respectively. It means that the GA 4024 hydrogel has a low sagging tendency after applying shear stress, but its structure regeneration is fast.

The viscosity decrement is higher, but the structure regeneration is faster in the case of high reaction temperature. In the case of malic acid, the viscosity decrement is lower, and the structure regeneration is faster than in the case of glutaric acid (Table 2).

**Table 2:** Viscoelastic Properties of Chitosan Hydrogels. SR: Structure Regeneration

	1st interval		2nd interval		3rd interval	
sample	SR (%)	SR (Pa·s/min)	SR (%)	SR (Pa·s/min)	SR (%)	SR (Pa·s/min)
MA 2524	94.83	1.48 × 10^–4^	89.65	1.48 × 10^–4^	84.48	1.48 × 10^-4^
MA 4024	93.56	1.36 × 10^–4^	87.11	1.36 × 10^–4^	80.67	1.36 × 10^–4^
MA 5024	95.18	1.43 × 10^–4^	90.37	1.43 × 10^–4^	85.55	1.43 × 10^–4^
GA 2524	94.59	9.84 × 10^–5^	92.83	1.08 × 10^–4^	91.06	1.10 × 10^–4^
GA 4024	85.42	9.84 × 10^–5^	84.28	1.18 × 10^–4^	83.13	1.18 × 10^–4^
GA 5024	79.23	1.10 × 10^–4^	75.15	2.45 × 10^–4^	74.30	2.81 × 10^–4^

The viscosity curves of hydrogels and all other rheological parameters of all samples can be found in Supporting Information material, Figures S3, S4 and Tables S2, S3.

### TGA and DSC Analyses of Chitosan Hydrogels

**Figure 6:**
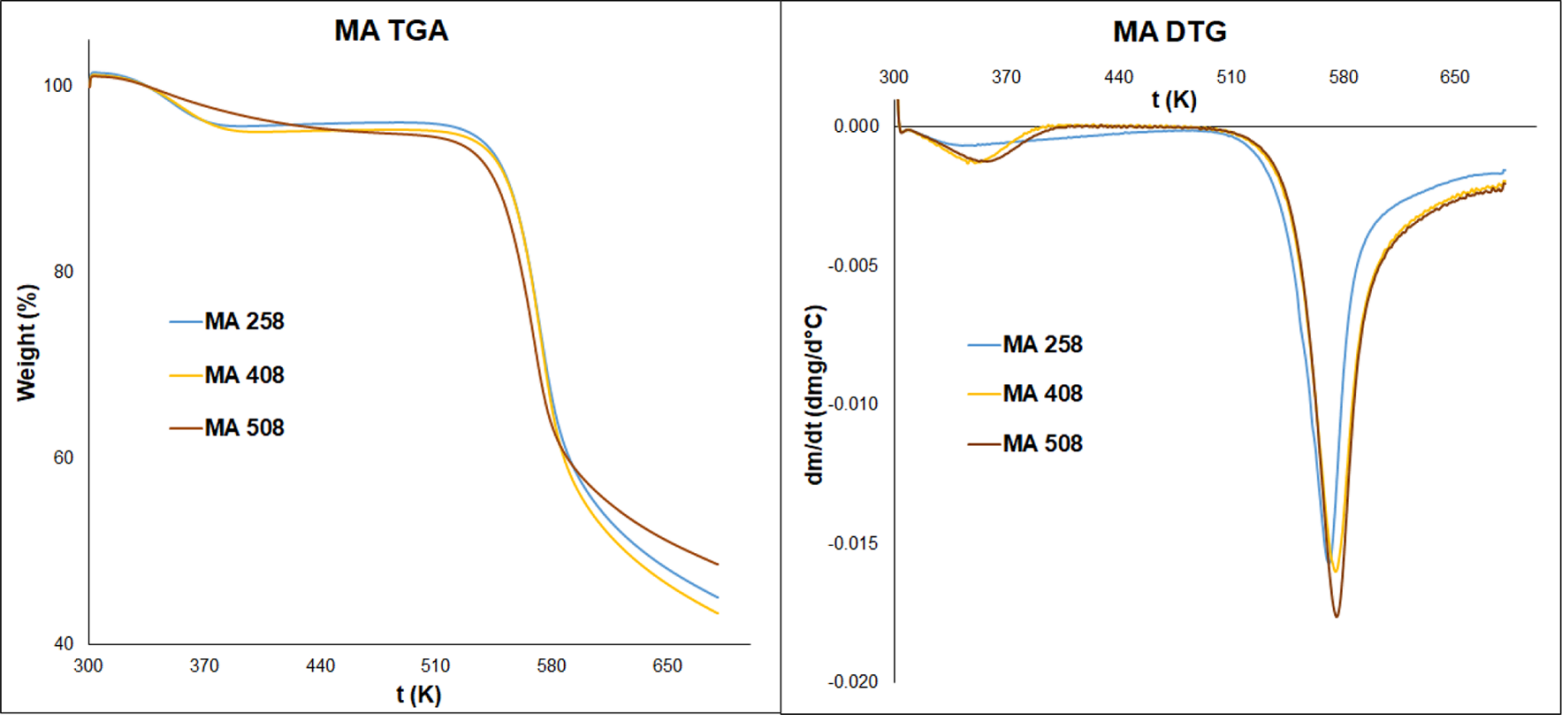
TGA and the derivative curves of chitosan hydrogels cross-linked with malic acid.

**Figure 7:**
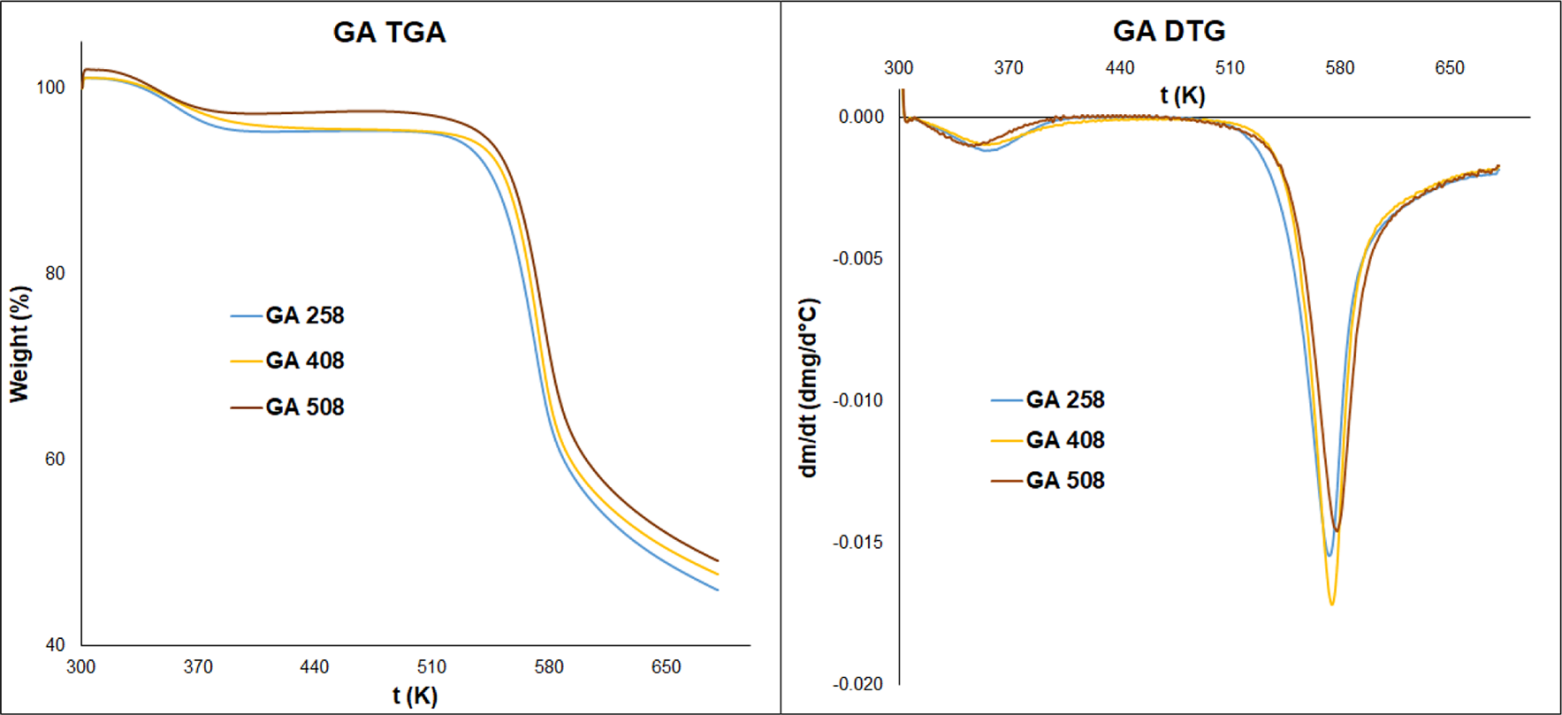
TGA and the derivative curves of chitosan hydrogels cross-linked with glutaric acid.

The thermal properties of hydrogels were examined with thermogravimetric analysis. Figures 6 and 7 show the thermogravimetric analysis (TGA) curves of hydrogels. The hydrogels lost their weight in two stages. Around 353.15 K, the weight loss is between 2.15 and 7.63%. The second stage starts at 513.15 K and reaches a maximum of around 573.15 K with 30.07–49.89% weight loss. The first stage is assigned to the loss of water and the second one is assigned to the thermal decomposition and depolymerization of chitosan chains (Table 3).^24,25^ It is assumed that the predrying procedure causes the low water content of our samples.

**Table 3:** TGA Results of Hydrogels

sample	*T*_1_ (K)	weight loss (%)	*T*_2_ (K)	weight loss (%)
MA 253	357.45	5.61	570,15	41.60
MA 258	357.50	5.66	569,70	42.78
MA 2524	357.46	5.47	569,70	42.15
MA 403	351.16	5.81	579,31	39.25
MA 408	351.15	5.61	579,92	39.16
MA 4024	351.30	5.94	579,38	37.05
MA 503	348.80	2.15	583,26	31.09
MA 508	348,48	2.22	586,92	30.07
MA 5024	348,24	2.41	588,20	31.42
GA 253	360,35	5.81	567,65	49.89
GA 258	360,55	5.83	567,65	40.02
GA 2524	360,35	5.85	568,30	34.98
GA 403	356,42	7.63	574,45	37.11
GA 408	357,15	7.60	574,45	38.56
GA 4024	357,15	7.14	574,70	37.66
GA 503	350,20	3.76	578,10	35.08
GA 508	346,77	2.31	578,25	35.05
GA 5024	349,75	3.52	579,20	34.18

In the first stage of TGA curves, their shapes and the peaks are different, where the water loss of hydrogels happens. It is well known from the literature that the hydration property of chitosan and its hydrogels and the water-holding capacity depend on the molecular structure. Because of the cross-linking in hydrogels, the molecular structure changes.^26^ Rueda and his colleagues determined that in the case of pure chitosan, the water molecules could bind to both hydroxyl and amino groups.^27^ The results show that increasing the reaction time and temperature, lower temperature values needed for the water removal of hydrogels, and the value of weight loss will also be lower (Table 3). Furthermore, it indicates that in the case of densely cross-linked hydrogels, where most of the amino groups are transformed into amide groups, the water molecules bind weakly.

In the second stage, the degradation temperature of chitosan increases if the reaction time and temperature increase. The degradation temperature of malic acid cross-linked hydrogels is lower than that of glutaric acid cross-linked samples. The increment of degradation temperature also confirms the formation of densely cross-linked hydrogels. This phenomenon indicated that the thermal stability of hydrogels increases if their cross-linking density increases. From the DSC results, any further information cannot be drawn about the thermal properties of hydrogels. The DSC curves are provided in the Supporting Information material (Figures S5 and S6).

### Swelling Properties of Hydrogels

Based on Flory’s theory, the water uptake is related to the extent of a cross-link of a polymer gel network;^28^ the higher water uptake indicates a lower cross-linking density of a polymer gel.^29^

**Figure 8:**
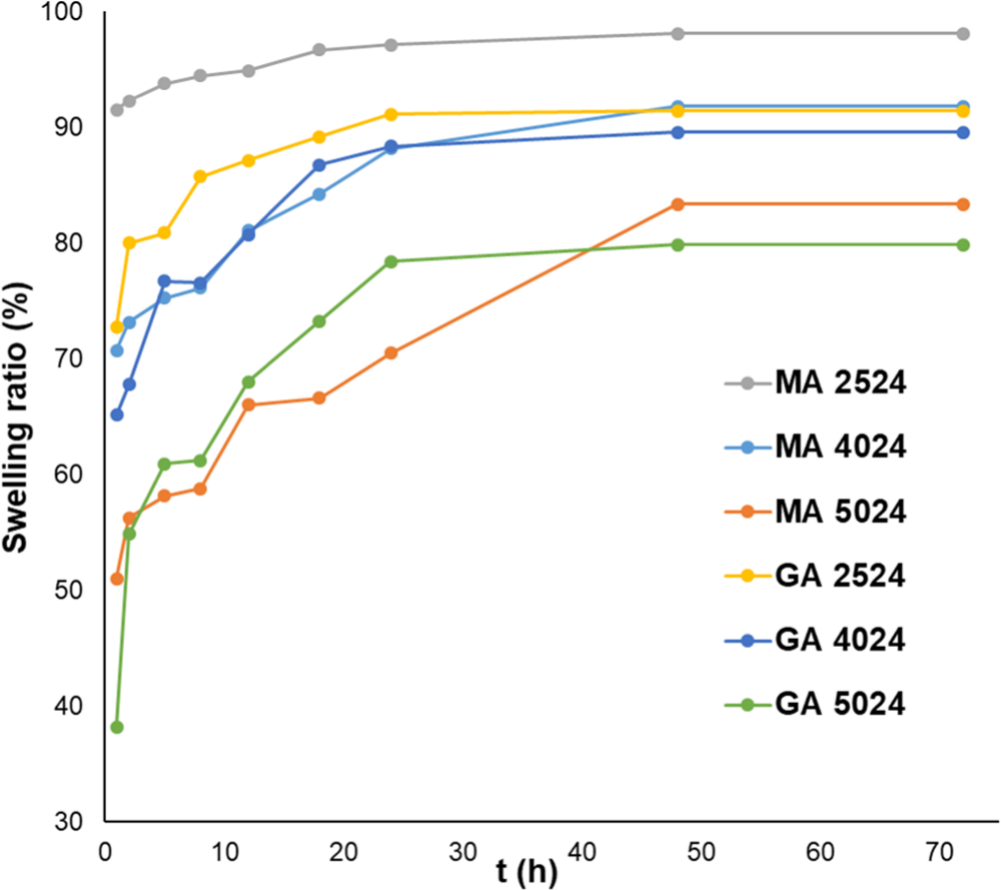
Swelling kinetics of chitosan hydrogels. MA: hydrogels cross-linked with malic acid. GA: hydrogels cross-linked with glutaric acid.

**Figure 9:**
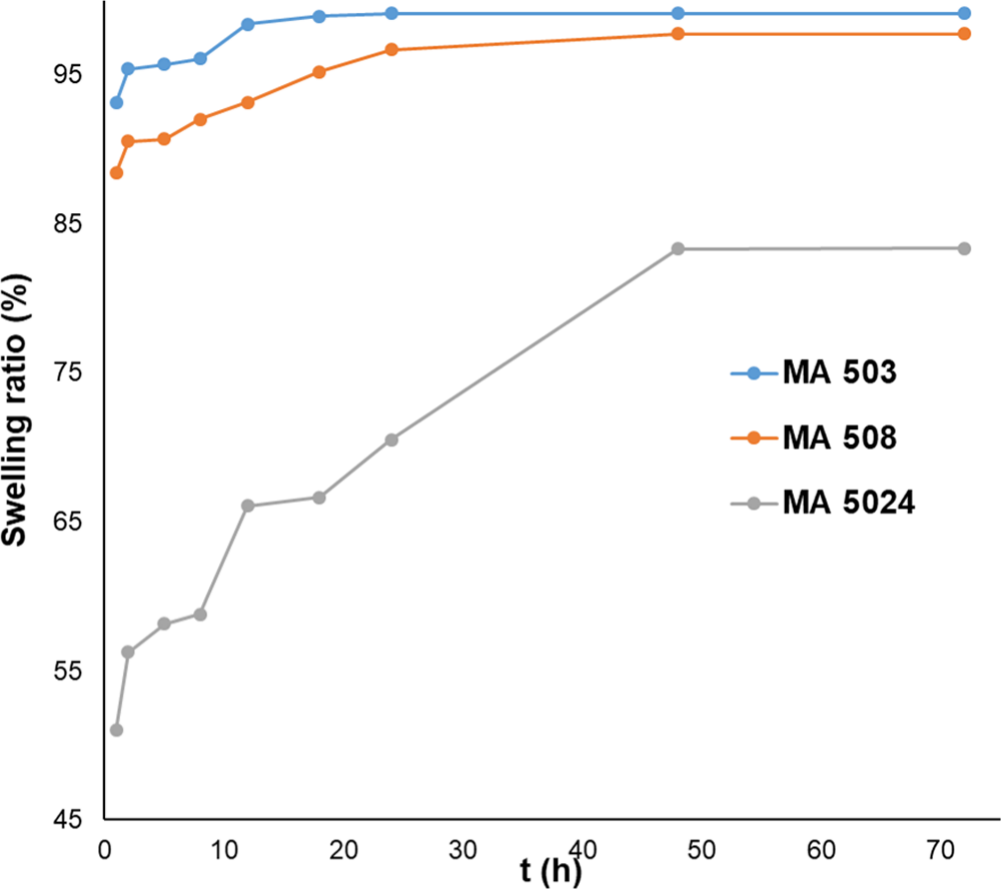
Swelling kinetics of chitosan hydrogels cross-linked with malic acid.

The curves of swelling kinetics of different chitosan hydrogels can be seen in Figure 8. The results clearly show that the reaction temperature of hydrogel preparation influences the swelling characteristics. With increasing reaction temperature, the swelling becomes slower, and the ratio becomes lower at each time point, in the case of both glutaric and malic acid. The reaction time also has a similar effect on swelling properties. Figure 9 shows three different gels cross-linked with malic acid at constant reaction temperature (313.15 K). With increasing reaction time, the swelling of hydrogels becomes slower, and the swelling ratio becomes lower. This tendency can be observed at each reaction temperature, for both glutaric and malic acid. This phenomenon indicates a densely cross-linked hydrogel formation with increasing reaction time and temperature.

The values of the equilibrium water content (EWC) can be found in Table 4. The weight of swollen samples did not change significantly after 48 h, so this time point is considered the equilibrium point of water uptake. The data suggest that the cross-linking density increases with increasing reaction time and temperature. In the case of the malic and glutaric acid cross-linked hydrogels, the difference between the EWC values can be observed at the same reaction temperature and time. The EWC of glutaric acid cross-linked hydrogels is lower than that of malic acid cross-linked ones. For example, the EWC is 98.13% in MA 2524 and 91.44% in the case of GA 2524. Assuming this phenomenon can be caused by the denser structure of glutaric acid cross-linked hydrogels and the cross-linkers’ different hydrophilic properties. The malic acid cross-linked hydrogels contain hydroxyl functional groups in the cross-linker, making their structure more hydrophilic than that of glutaric acid gross-linked samples.

**Table 4:** Equilibrium Water Content (ECW, %) of Chitosan Hydrogels^a^

sample	EWC (%)	sample	EWC (%)
MA 253	99.15	GA 253	99.20
MA 258	98.42	GA 258	98.57
MA 2524	98.13	GA 2524	91.44
MA 403	96.93	GA 403	97.00
MA 408	97.98	GA 408	94.58
MA 4024	91.84	GA 4024	89.58
MA 503	97.78	GA 503	94.25
MA 508	97.28	GA 508	90.36
MA 5024	83.36	GA 5024	79.90

a The equilibrium water content is calculated after 48 h of swelling.

### Characterization of API-Loaded Hydrogels

These experiments aimed to prove that the APIs can be adsorbed in hydrogels in the desired amount, which is enough to inhibit the binding of COVID-19 spike protein and ACE2 receptors on the nasal mucosa. Tin-Yun Ho and his colleagues established the IC_50_ value for emodin,^11^ which determines the quantity required to inhibit the interaction between S protein and ACE2 at 50%. Based on their research, the required concentration of emodin is 200 μmol·dm^–3^. Zhang and his research group examined the binding activity of glycyrrhizic acid to S protein of SARS-CoV-2 and ACE2 receptors; the measured IC_50_ concentration is 22 μ μmol·dm^–3^.^30^ Although Cheng et al. described that baicalin also has a high binding capacity for the ACE2 receptor,^31^ its IC_50_ value has been determined by Deng et al., which is 2.24 μmol·dm^–3^.^32^

The maximum adsorption capacities of hydrogels for different APIs are presented in Table 5. It can be concluded from the results that the maximal achievable API content of hydrogels is an order of magnitude higher than their IC_50_ values. For example, the GA 258 sample has a less emodin content, 8.78 × 10^–4^ mol/g, which is about four times greater than the concentration needed to inhibit ACE2 receptor binding of COVID spike protein. Similarly, the GA 408 sample has a low glycyrrhizic acid content (8.94 × 10^–3^ mol/g), and the MA 408 hydrogel has a low baicalin content (2.32 × 10^–3^ mol/g), but these values also exceed the IC_50_ concentrations of glycyrrhizic acid and baicalin.

**Table 5:** API Adsorption Capacity of Different Hydrogels^a^

hydrogel	API	*n*_ads max_(mol/g)	hydrogel	API	*n*_ads max_(mol/g)
MA 258	emodin	1.14 × 10^–3^	GA 258	emodin	8.78 × 10^–4^
MA 408		1.13 × 10^–3^	GA 408		8.82 × 10^–4^
MA 508		1.13 × 10^–3^	GA 508		9.84 × 10^–4^
MA 258	glycyrrhizic acid	9.42 × 10^–3^	GA 258	glycyrrhizic acid	9.04 × 10^–3^
MA 408		9.37 × 10^–3^	GA 408		8.94 × 10^–3^
MA 508		9.52 × 10^–3^	GA 508		9.24 × 10^–3^
MA 258	baicalin	2.49 × 10^–3^	GA 258	baicalin	2.51 × 10^–3^
MA 408		2.32 × 10^–3^	GA 408		2.44 × 10^–3^
MA 508		2.41 × 10^–3^	GA 508		2.41 × 10^–3^

a The *n*_ads__max_ value determines the adsorption capacity; namely, the maximum API amount (mol) can be adsorbed on 1 g chitosan hydrogel.

The adsorption isotherms of hydrogels can be found in Supporting Information material (Figures S10–S12).

**Figure 10:**
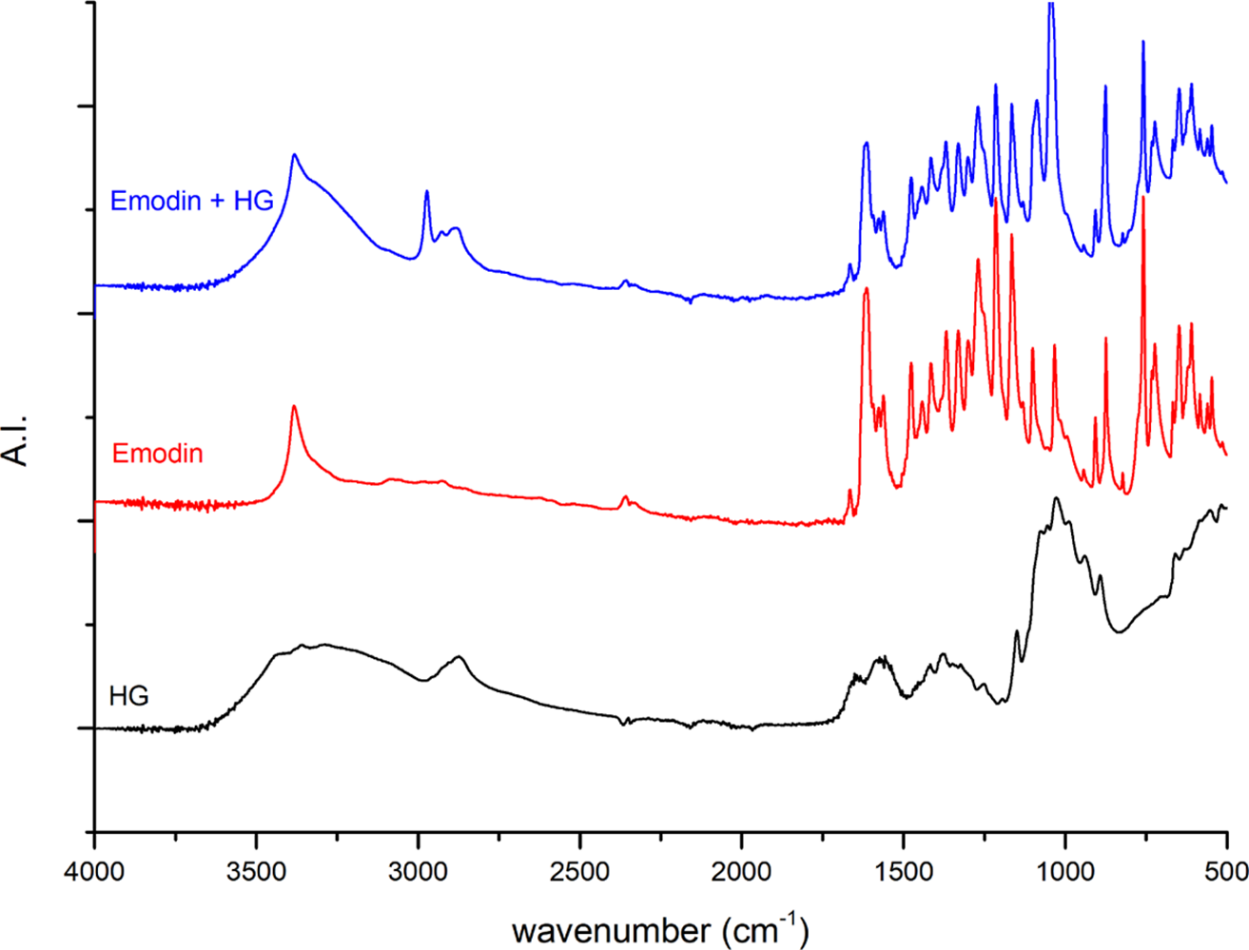
FTIR spectra of the GA 408 hydrogel (HG), emodin, and emodin-loaded GA 408 hydrogel.

**Figure 11:**
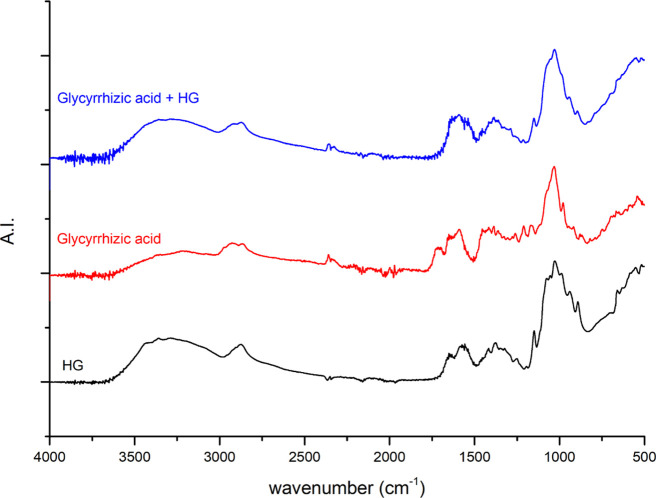
FTIR spectra of the GA 408 hydrogel (HG), glycyrrhizic acid, and glycyrrhizic acid-loaded GA 408 hydrogel.

**Figure 12:**
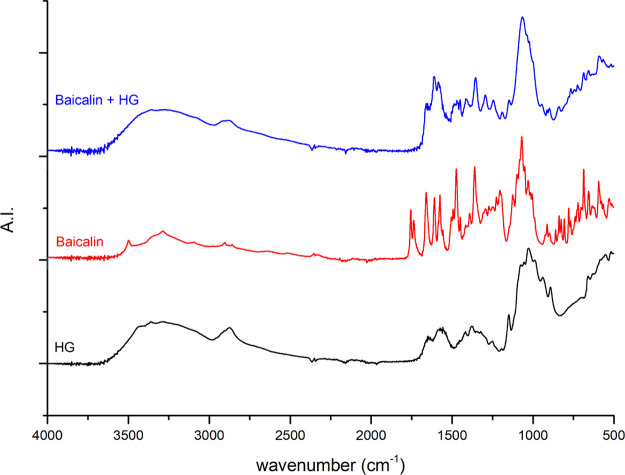
FTIR spectra of the GA 408 hydrogel (HG), baicalin, and baicalin-loaded GA 408 hydrogel.

Figures 10–12 show the FTIR spectra of the GA 408 hydrogel, the different APIs, and the API-loaded GA 408 hydrogels. The results indicate successful API adsorption on hydrogels because characteristic peaks for hydrogels and API can be observed in each spectrum of API-loaded hydrogels.

### Results of Cytotoxicity Measurements

The cytotoxicity of different hydrogels was also tested on the HEK 293 cell line with cell viability measurements. The chitosan-based nasal delivery systems are generally considered biocompatible, but based on previous studies and our results, the cytotoxic effect of hydrogels is found to be highly concentration-dependent. The results show that the pure and API-loaded chitosan hydrogels at a 0.1 mg/mL concentration value is non-toxic; the glutaric acid cross-linked samples show lower cytotoxicity than the malic acid cross-linked hydrogels. Using the hydrogels at a 10.0 mg/mL concentration, the samples show higher toxicity than the positive control. The detailed results can be found in the Supporting Information Material, Figures S10 and S11.

### In Vitro Release Study of Hydrogels

**Figure 13:**
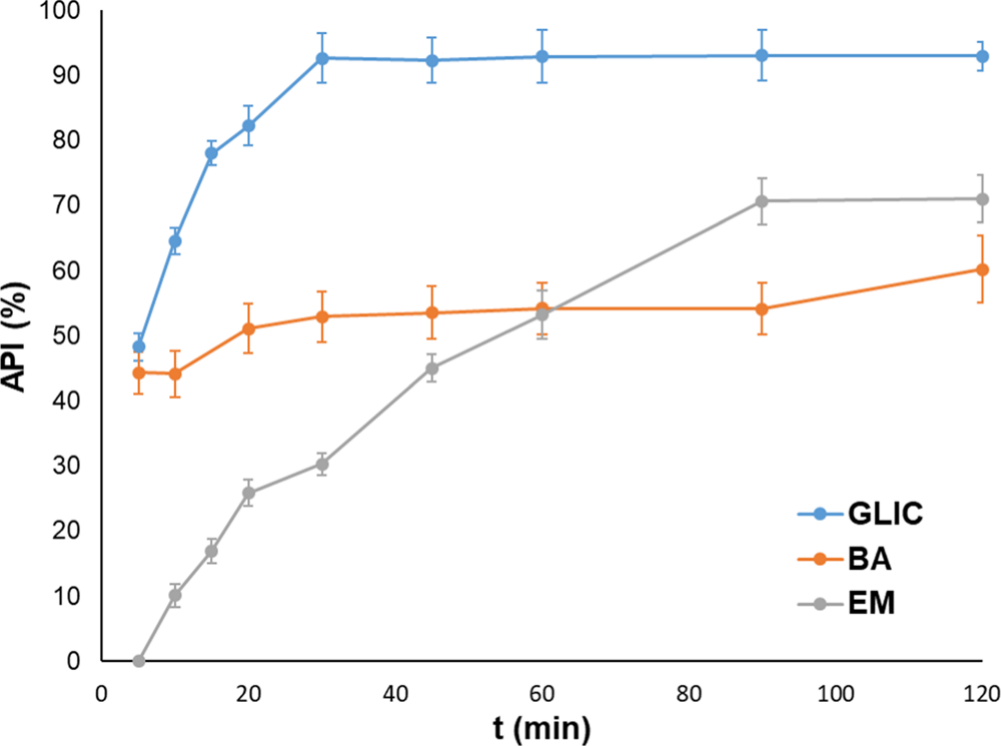
Glycyrrhizic acid, baicalin, and emodin release profiles from the MA 408 hydrogel. GLIC: glycyrrhizic acid, BA: baicalin, and EM: emodin.

**Figure 14:**
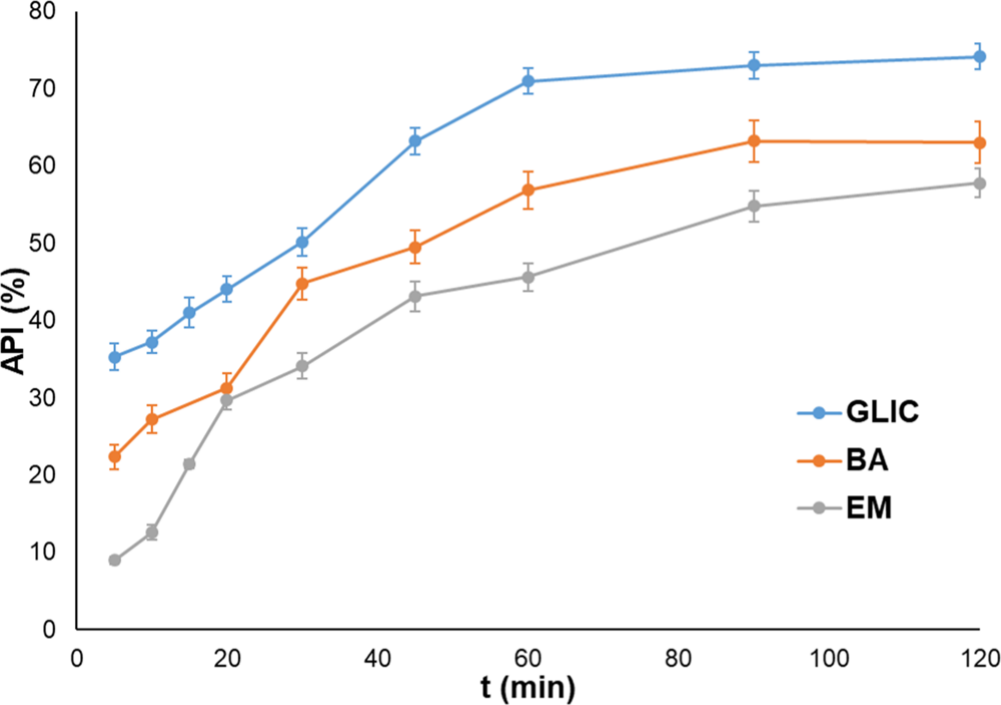
Glycyrrhizic acid, baicalin, and emodin release profiles from GA 508 hydrogel. GLIC: glycyrrhizic acid, BA: baicalin, and EM: emodin.

In Figures 13 and 14, the release profiles of API-loaded chitosan hydrogels can be seen. In the case of hydrogels cross-linked with malic acid (MA 408), the cumulative API amounts after 2 h are higher than those of hydrogels cross-linked with glutaric acid (GA 408). The cumulative glycyrrhizic acid amount is 92.99 and 74.13% from MA 408 and GA 408, respectively. These values for baicalin and emodin are 60.23 and 63.00, and 71.08 and 57.79%, from MA 408 and GA 408, respectively. These differences in the released API amount between the MA 408 and GA 408 hydrogels can be caused by their different hydrogel densities; from the densely cross-linked GA 408 hydrogel, the API release can be slower and lower than that from the slightly cross-linked MA 408 hydrogels.

### Determination of Mucoadhesive Properties of API-Loaded Hydrogels

**Figure 15:**
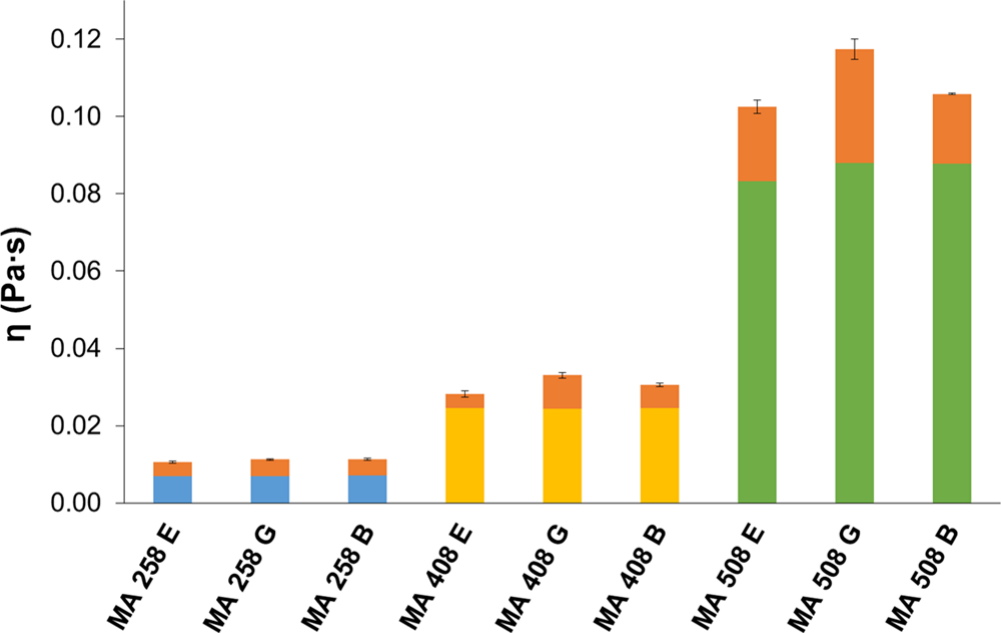
Results of mucoadhesivity test of API-loaded hydrogels cross-linked with malic acid. EM: emodin. GLY: glycyrrhizic acid. BA: baicalin. The orange columns represent the increment of viscosity caused by the interaction of hydrogels with mucin.

**Figure 16:**
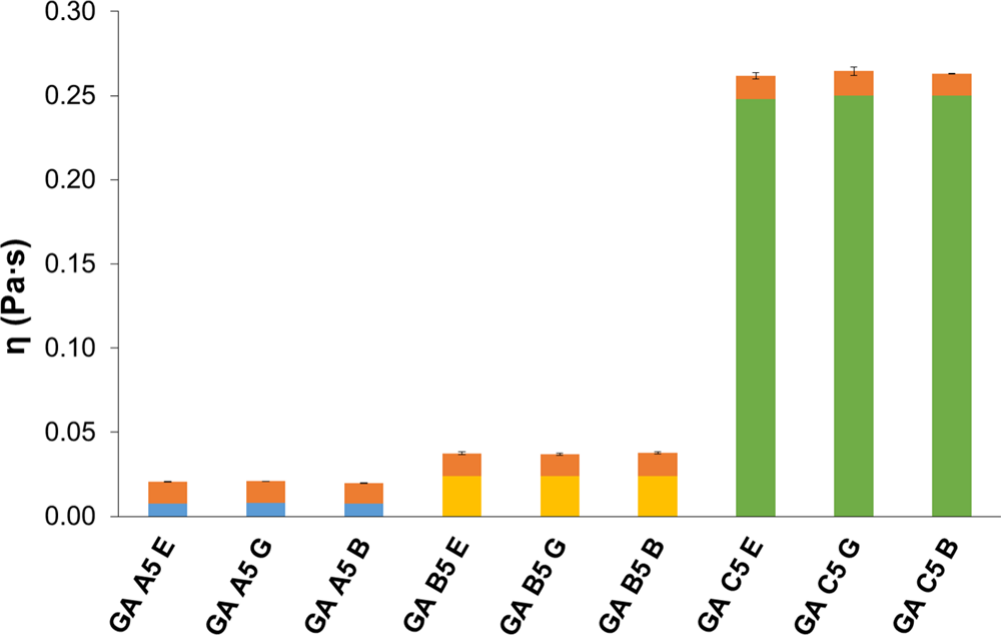
Results of the mucoadhesive test of API-loaded hydrogels cross-linked with glutaric acid. E: emodin. G: glycyrrhizic acid. B: baicalin. The orange columns represent the increment of viscosity caused by the interaction of hydrogels with mucin.

Several research groups have described before that the chitosan and chitosan hydrogels show mucoadhesive properties; therefore, they can be suitable for nasal administration of different APIs.^17^ Furthermore, the results show that the viscosity of different API-loaded hydrogels increased if they were mixed with mucin dispersion, which confirms their mucoadhesive properties (Figures 15, 16).

**Figure 17:**
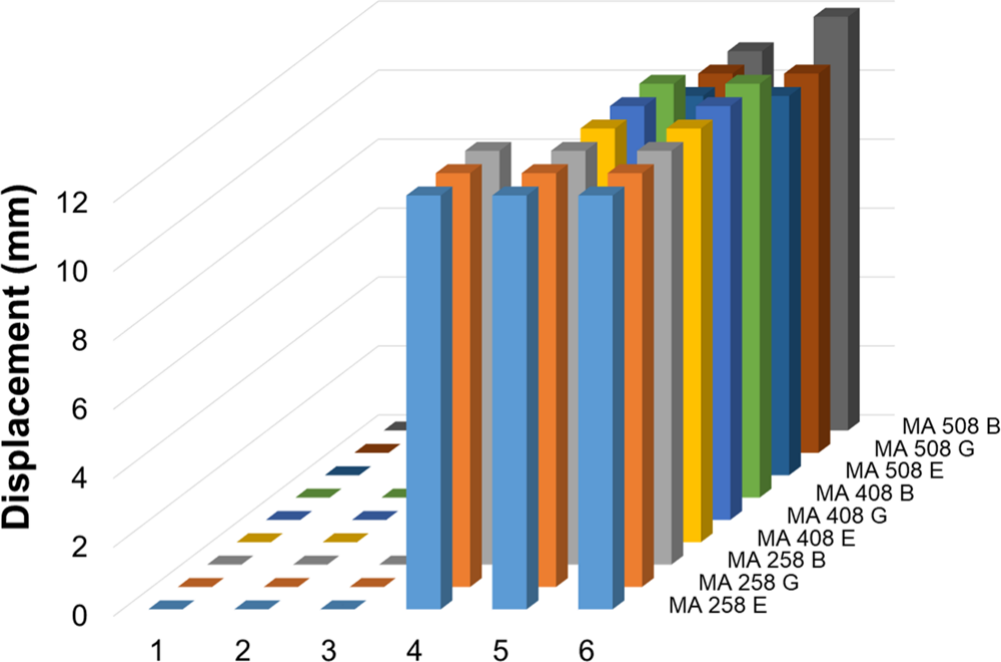
Mucoadhesion profiles of different chitosan hydrogels cross-linked with malic acid determined using the displacement method. E: emodin. G: glycyrrhizic acid. B: baicalin.

**Figure 18:**
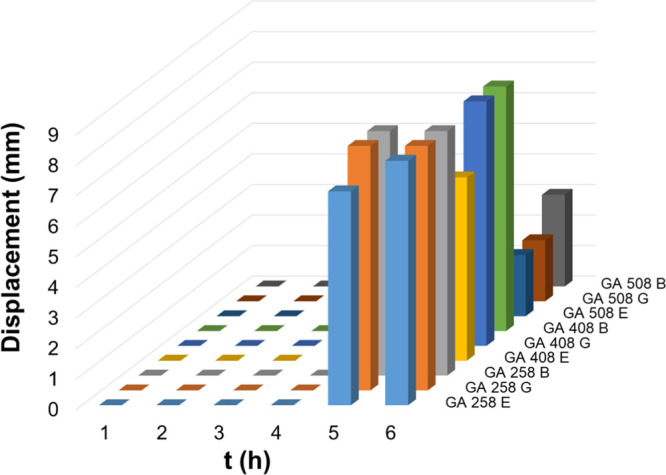
Mucoadhesion profiles of different chitosan hydrogels cross-linked with glutaric acid determined using the displacement method. E: emodin. G: glycyrrhizic acid. B: baicalin.

The mucoadhesive properties were also tested using the displacement method. The displacement (downwards movement of the hydrogel) in mm was measured hourly for up to 6 h (Figures 17 and 18). All hydrogels show high bioadhesive properties: in the case of malic acid, cross-linked hydrogel displacement of samples could not be observed until 3 h, and in the case of glutaric acid, cross-linked hydrogels until 4 h. However, the densely cross-linked hydrogels (GA C5 and GA B5 samples) show the highest bioadhesive property; the displacement values are only 2–3 mm after 6 h.

## Conclusions

This study aimed to prepare mucoadhesive, chemically cross-linked chitosan hydrogels with malic and glutaric acid, without adding any other excipients, solubilizing agents, or catalysts, and examine their interaction with some natural ACE2 receptor inhibitors. They are prepared to use in a nasal formulation to reduce the risk of COVID-19 infection.

The results of IR spectroscopy confirmed that under appropriate reaction conditions, covalent acetamide bonds formed between the free amino groups of chitosan and carboxyl groups of a dicarboxylic acid. However, in the IR spectra, the characteristic peak of free carboxyl groups cannot be observed. It means that both carboxyl groups take part in the acetamide bond formation building the chitosan hydrogel structure.

Based on the results of conductometric titration, the number of free amino groups in chitosan hydrogels decreased compared to that of chitosan. The cross-linking degree was calculated from the number of free amino groups of hydrogels and chitosan. By increasing the reaction time and temperature, the cross-linking degree increased. Higher cross-linking density can be observed using glutaric acid as a cross-linking agent than malic acid at each reaction temperature and time point. The glutaric acid cross-linked hydrogels contain a higher number of cross-links, which means that these hydrogels have a stronger, denser gel network. The flow, thermal, and swelling properties are in good agreement with the results presented above. All hydrogels show Bingham plasticity and thixotropy. At a higher cross-linking degree, the Bingham yield point and viscosity values are higher, indicating the formation of a densely cross-linked hydrogel. The viscosity values are lower than 500 mPa·s in each case, making hydrogels suitable for nasal formulation. The thixotropic behavior is also related to the relative cross-linking degree: the thixotropy is more pronounced if the hydrogel has a higher cross-linking degree. The results show the difference between malic and glutaric acid cross-linked hydrogels; when using glutaric acid as a cross-linking agent, the Bingham yield point and viscosity values are higher, and their thixotropic behavior is more pronounced, which also confirms the formation of a denser, more robust hydrogel structure. The increment of the cross-linking degree is also related to the swelling properties: the densely cross-linked hydrogels can uptake less water, and their swelling is slower than samples with lower cross-linking density. Besides the cross-linking density, the hydrophilic property of the cross-linking chain also influences the swelling of hydrogels, and the malic acid cross-linked samples can uptake more water than glutaric acid cross-linked ones of the hydroxyl groups of the cross-linking chain.

As a summary of the characterization of chitosan hydrogels, it can be concluded that densely cross-linked hydrogels can be prepared with increasing reaction time and temperature, in the case of using both malic and glutaric acid. Using glutaric acid as a cross-linking agent at each reaction time and temperature resulted in a denser, stronger hydrogel.

Based on the literature, three different natural compounds that inhibit the interaction between the viral spike protein and ACE2 receptors in the nasal mucosa were chosen for the development of the formulation. The results showed that mucoadhesive hydrogels could adsorb a sufficient amount of the API necessary to reach the inhibitory concentration (*c* ≥ IC_50_). The release tests showed that the hydrogel density influences the amount of released API; the release is slower from the denser GA hydrogel samples. After 2 h, the IC_50_ amount can be reached.

Chemically cross-linked chitosan hydrogels can be prepared using a water solution of malic or glutaric acid under mild reaction conditions. The properties of API-loaded hydrogels are suitable for use as a nasal formulation. However, the virus-binding inhibition ability is yet to be tested.

## Materials and Methods

### Preparation of Hydrogels

The chitosan solubility was previously determined in malic acid or glutaric acid solutions, and the results are shown in the Supporting Information material (part 1, Figures S1 and S2). 5 g of chitosan was dissolved in 500 mL malic or glutaric acid (10 g/dm^3^), the concentration of dicarboxylic acids was 20 g/dm^3^. Chitosan MW: 100,000–300,000 Da, the degree of deacetylation ≥75% (Acros Organics, part of Thermo Fischer Scientific Inc., USA), dl-malic acid purity ≥99.0% (Acros Organics, part of Thermo Fischer Scientific Inc., USA), and glutaric acid purity ≥99.0% (Alfa Aesar, part of Thermo Fischer Scientific Inc., USA).

The reaction time was 24 h at constant reaction temperature (*t* = 298.15/313.15/323.15 K). Samples were taken after 1.5, 3, 4.5, 6, 8, and 24 h. The sample was added to 10-fold volume demineralized water (Ministill P12 water purifier system, BWT AG, Austria) to decrease the chitosan solubility and precipitate the hydrogels. The samples were centrifuged at 5000 m^–1^ for 2 min at 288.15 K. Then, the supernatant was removed, and the gel was washed with demineralized water and centrifuged again. The washing procedure was repeated until the pH = 7.2–7.4 was reached. Then, the gel was swollen in demineralized water for 24 h and stored in the fridge (277.15–281.15 K) until further use. The sample nomenclature with comments can be seen in Table 6.

**Table 6:** Nomenclature of Hydrogel Samples

chitosan hydrogels cross-linked with malic acid			chitosan hydrogels cross-linked with glutaric acid		
sample	*T*_reaction_ (K)	*t*_reaction_ (h)	sample	*T*_reaction_ (K)	*t*_reaction_ (h)
MA 251.5	298.15	1.5	GA 251.5	298.15	1.5
MA 253		3	GA 253		3
MA 254.5		4.5	GA 254.5		4.5
MA 256		6	GA 256		6
MA 258		8	GA 258		8
MA 2524		24	GA 2524		24
MA 401.5	313.15	1.5	GA 401.5	313.15	1.5
MA 403		3	GA 403		3
MA 404.5		4.5	GA 404.5		4.5
MA 406		6	GA 406		6
MA 408		8	GA 408		8
MA 4024		24	GA 4024		24
MA 501.5	323.15	1.5	GA 501.5	323.15d	1.5
MA 503		3	GA 503		3
MA 504.5		4.5	GA 504.5		4.5
MA 506		6	GA 506		6
MA 508		8	GA 508		8
MA 5024		24	GA 5024		24

### FT IR Spectroscopy

FTIR spectral analysis was performed at ambient temperature on a Thermo Nicolet 380 FT IR spectroscope (Thermo Fisher Scientific, USA) in attenuated total reflectance mode (Smart Orbit diamond ATR). Data were collected in the 4000–400 cm^–1^ wavenumber range, and each spectrum was averaged over 60 scans with a 4 cm^–1^ resolution. The air as a background was taken before each sample run. The samples for analysis were prepared as follows: first, the hydrogels were centrifuged at 5000 m^–1^ for 3 min (15 °C), dried at 313.15 K for 24 h in a drying chamber (Binder B053, Binder GmbH, Germany), and then stored in a desiccator (JEOL EM-DSC10E, JEOL Ltd., Japan) until the measurements were carried out.

### Determination of the Number of Free Amino Groups

Determination of the number of free amino groups of chitosan and chitosan hydrogels was performed with conductometric titration as described before by Fonseca et al.^33^ Briefly, chitosan and chitosan hydrogel solutions were prepared by the dissolution of a known mass of the sample (∼0.05 g) in 10 mL of 0.01 mol·dm^–3^ HCl solution at room temperature (298.15 K). Deionized water was used for the preparation of all solutions to avoid the presence of external ions. After the addition of 25 mL water, conductometric titration was carried out with 0.025 mol·dm^–3^ NaOH solution using a Mettler Toledo Seven2Go S3 conductivity meter (Mettler Toledo GmbH, Giessen, Germany) and an InLab 738-ISM sensor (Mettler Toledo GmbH, Giessen, Germany). All titrations were carried out in quadruplicate.

The number of moles of aminated groups (*n*_A_) is given by the following equation
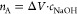
where Δ*V* = Δ*V*_NaOH_,_f_ – Δ*V*_NaOH_,_i_ and *c*_NaOH_ is the concentration of NaOH solution used for titration.

### Rheological Studies

The rheological analysis of hydrogels was performed with a rotational viscometer (RheoLab QC, Anton Paar, Anton Paar GmbH, Graz, Austria) using a concentric cylinder measuring system (CC27-SN16152) and the RheoPlus software. To record the flow and viscosity curves, the data of shear stress and apparent viscosity were collected as a function of shear rate. The rotational speeds were varied between 20 and 1200 s^–1^ for the upward curves, and between 1200 and 20 s^–1^ for the downward curves. The duration of measurements was 600 s with 50 measurement points in both cases. The relation between shear stress and shear rate was analyzed with the Bingham plasticity mathematical model^34,35^
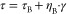
where τ is the yield stress, τ_B_ is the Bingham yield point (Pa), η_B_ is the Bingham viscosity (Pa·s), and γ is the shear rate (s^–1^). Time-dependent flow properties were also measured, the measurement consists of two steps. In the first step, the viscosity changes of hydrogels were investigated at a constant shear rate (100 min^–1^), and the duration of experiments was 10 min. In the second step, the samples were left to relax up to 20 min. These two measurement steps were repeated three times.

To quantify the thixotropy of hydrogels, the hysteresis area method has been performed: the measurement settings of these experiments was the same as in the case of flow curve measurements, where the upward and downward ramps are repeated 10 times. The hysteresis area indicates the degree of system destructuration, higher values for thixotropic area indicate a higher thixotropy. The area of the hysteresis loop (*A*_hys_) is the surface between the upward curve (*A*_upw_) and the downward curve (*A*_dw_)
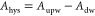


All rheological parameters were evaluated using RheoPlus software.

### TGA and DSC

TGA and differential scanning calorimetry (DSC) measurements were carried out simultaneously with a Mettler Toledo DSC 821e instrument. All analyses were performed with a 8 mg sample in alumina sample holders under a nitrogen atmosphere between 298.15 and 673.15 K. The experiments were run at a scanning rate of 10 K/min. STARe Evaluation Software was used for data collection and calculation of the first derivative of curves. All samples were predried at 313.15 K for 24 h and stored in a desiccator (JEOL EM-DSC10E, Jeol Ltd., Japan) until the measurements were carried out.

### Swelling Kinetics Experiments

Swelling kinetics experiments were conducted in phosphate-buffered saline (PBS, pH = 7.40) at room temperature (298.15 K). For the buffer preparation, the following chemicals were used: NaCl (high purity, VWR Chemicals Ltd., Hungary), KCl (purity 99%–100.5%, VWR Chemicals Ltd., Hungary), Na_2_HPO_4_·2H_2_O (AnalaR NORMAPUR, purity ≤99.0%, VWR Chemicals Ltd., Hungary), and KH_2_PO_4_ (purity ≤99.0%, VWR Chemicals Ltd., Hungary).

The hydrogel samples were centrifuged at 5000 m^–1^ for 2 min and dried at 313.15 K for 24 h. The dried samples were weighted (*w*_0_) and placed in glass bottles in PBS buffer solution. After each interval, the PBS solution was removed, and the weight of swollen samples (w_*t*_) was measured. The buffer was freshened after every interval. The samples were weighted after 1, 2, 5, 8, 12, 18, 24, 48, and 72 h. The ratio of swelling at time *t* was calculated using the following equation
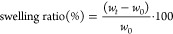


ECW was also calculated for each sample: we consider the that swelling ratio is equal to ECW, if the weight of the swollen hydrogel did not change in time significantly.

### Determination of API Adsorption Capacity of Hydrogels

For the experiments, the following chemicals were used: emodin (pur. ≥98.5%, Alfa Aesar GmbH, Germany), baicalin (pur. ≥97.0%, Cayman Chemicals Company, USA), glycyrrhizic acid (pur. ≥98.0%, Acros Organics part of Thermo Fischer Scientific Inc., USA), demineralized water (Ministill P12 water purified system, BWT AG, Austria), and absolute ethanol (AnalR NORMAPUR; purity ≥99.8%; VWR Chemicals Ltd., Hungary).

The method used for the determination of adsorption capacity was the same as in the case of the three APIs, except for the reaction medium, because of their different solubility. The reaction medium was absolute ethanol in the case of emodin and baicalin, and demineralized water in the case of glycyrrhizic acid. In the experiments, where absolute ethanol was used, first the hydrogel samples were centrifuged at 5000 m^–1^ for 2 min, dried at 313.15 K for 24 h, and swollen in absolute ethanol for 24 h.

1.0 g hydrogel sample was mixed with an adequate amount of API solution and stirred with 800 m^–1^ at room temperature (298.15 K) for 24 h. Then, the samples were centrifuged at 5000 m^–1^ for 2 min at 288.15 K, and the supernatants were collected and the API concentration was measured with a UV–vis spectrophotometer (Jasco V-550 UV/vis Spectrophotometer; Jasco Inc., USA). The wavelengths of the adsorption maximum of APIs were as follows: emodin, λ_max_ = 439 nm; baicalin, λ_max_ = 317 nm; and glycyrrhizic acid, λ_max_ = 257 nm. Nine adsorption points were measured in each case. The adsorbed amounts of API in hydrogels (*n*_ad_ [mol/g hydrogel]) were calculated from the difference of the measured API (*n*_m_ [mol]) and its molar amount in the supernatant (*n*_s_ [mol]) divided by the weight of hydrogel (*m*_hg_ [g])
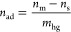


The adsorption capacity of the gels (*n*_ad_^max^) was determined graphically from their adsorption isotherm.

### Cytotoxicity Test and Analysis

#### Cell Culture

Human embryonic kidney 293 cells (HEK-293) were maintained in Dulbecco’s modified Eagle’s medium (DMEM) (Lonza) supplemented with 10% fetal bovine serum (FBS, Euroclone), penicillin–streptomycin (Lonza), l-glutamine (Lonza), HEPES buffer (Lonza), non-essential amino acids (Lonza), and β-mercapto-ethanol (Sigma), at 37 °C, in a humidified atmosphere, containing 5% CO_2_. For the cell viability test, 3 × 10^4^ cells were seeded in 24-well plates in 1 mL of complete DMEM (cDMEM) in triplicate. Also, HEK-293 cells were treated with 3% dimethyl sulfoxide (DMSO) (Santacruz Biotech) and used as positive controls in the apoptosis assay.

#### Cytotoxicity Experiments

After 24 h of incubation, the cells were washed with PBS (Lonza) and trypsinized (Lonza) until detachment. Enzymatic digestion was stopped using equal volumes of cDMEM containing 10% FBS and centrifuged for 5 min at 400 g at room temperature. Cell pellets were washed with PBS and after a second centrifugation, cells were resuspended in 100 μL AnnexinV Binding Buffer (10 mM HEPES, 140 mM NaCl, 25 mM CaCl_2_, pH 7.4). Next, the Annexin V-Alexa 488 conjugate (Thermo, cat. no. A13201) was added to each cell suspension and incubated for 15 min in the dark, at room temperature. Finally, 7-AAD viability staining solution was added to the samples (Thermo, cat. no. 00-6993-50) and kept on ice until data acquisition. A BD FACS Canto II flow-cytometer (Becton Dickinson) was used for data acquisition at a medium flow rate and stopped at 10,000 events. Measurements were performed and analyzed with BD FACSDiva Software version 6.1.3.

#### In Vitro Release Studies

A 1 mg sample of hydrogel was placed into a vessel containing 40 mL PBS buffer, pH = 7.40, *t* = 310.15 K. The solution was stirred at 50 m^–1^ for up to 2 h. At predetermined time intervals, 2 mL of the release medium was taken and replaced by an equal volume of PBS buffer. Time intervals were 5, 10, 15, 20, 30, 45, 60, 90, and 120 min. The API content was determined with UV–vis spectroscopy, as it is described in part “determination of API adsorption capacity of hydrogels” before. All measurements were performed in triplicate.

### Determination of Mucoadhesive Properties of API-Loaded Hydrogels

#### Evaluation of Mucoadhesivity with Viscosity Measurements

Viscosity measurements were performed to determine the mucoadhesive interactions between mucin dispersions and hydrogels. According to Hassan and Gallo, the synergistic increase in viscosity can be observed when the polymer is mixed with mucin, which is an index of the strength of the mucoadhesive bond.^36^ They suggested the following equation to calculate the force of bioadhesion, which is widely used to determine the mucoadhesive potential of nasal formulations^17^
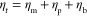
where η_*t*_ is the viscosity of the polymer–mucin mixture, η_m_ is the viscosity of the mucin dispersion, η_p_ is the viscosity of polymer solution, and η_b_ is the component of bioadhesion.

Kinematic viscosity values were determined with Ostwald-Fenske capillary viscometers. First, the mucin dispersion (10.0 m/m %) [bovine mucin from submaxillary glands, MP Biomedicals, Fisher Scientific GmbH, Schwerte, Germany] was freshly prepared with PBS buffer, and the dispersion was vigorously stirred for 12 h at room temperature. The API-loaded chitosan hydrogel samples were centrifuged at 5000 m^–1^ for 2 min, and then allowed to swell in PBS buffer overnight. The hydrogel samples were mixed with mucin dispersion under vigorous stirring for 15 min at room temperature, and the mixtures contained 1.0 m/m % hydrogels and 2.0 m/m % mucin. All viscosity measurements were performed in triplicate.

#### Evaluation of Mucoadhesivity with Calculation of the Displacement Factor

Mucoadhesion of different hydrogels was examined using the displacement method of Bertram and Bodmeier.^37^ A hot agar/mucin solution (1.0 and 2.0 w/w %, respectively) in PBS buffer (pH = 7.40) was cast on a glass plate (2 cm × 1 cm) and left to gel at 281.15 K for 3 h. Then, the gel was equilibrated for 2 h to the test conditions of 310.15 K and 78% relative humidity in an incubator (POL-EKO-Aparatura incubator, type CLN 53, POL-EKO-Aparatura sp.j. Poland). The hydrogels were placed on the top of the agar/mucin gel and inclined (angle 45°). The displacement in centimeter was measured hourly up to 6 h. The adhesion potential is inversely related to the displacement of the hydrogel.

## Supporting Information Available

Determination of chitosan solubility; results of calculation of the cross-linking degree and cross-linking efficacy; determination of flow properties; differential scanning calorimetry measurements of hydrogels; characterization of API adsorption of hydrogels; and cytotoxicity results (PDF)
