# Supplementary material for: Preparation and Characterization of ACE2 Receptor Inhibitor-Loaded Chitosan Hydrogels for Nasal Formulation to Reduce the Risk of COVID-19 Viral Infection
Source: ACS Omega. 2022 Jan 14;7(4):3240–53. doi: 10.1021/acsomega.1c05149 (PMC8790824; doi:10.1021/acsomega.1c05149)
Supplement: Supplementary file 1 — ao1c05149_si_001.pdf [file ao1c05149_si_001.pdf]

Preparation and characterization of ACE2 receptor inhibitor loaded chitosan hydrogels for nasal formulation to reduce the risk of COVID-19 viral infection

Barbara Vörös-Horváth[1], Pavo Živković[2], Krisztina Bánfai[3], Judit Bóvári-Biri[3], Judit Pongrácz[3], Gábor Bálint[1], Szilárd Pál[1], Aleksandar Széchenyi[1,2*] [1] Institute of Pharmaceutical Technology and Biopharmacy, University of Pécs, Faculty of Pharmacy, Rókus u. 2., H-7624 Pécs, Hungary, horvath.barbara@gytk.pte.hu, pal.szilard@gytk.pte.hu, szechenyi.aleksandar@gytk.pte.hu

[2] Department of Chemistry, Josip Juraj Strossmayer University of Osijek, Ulica cara Hadrijana 8/A, HR-31000 Osijek, Croatia; pzivkovic@kemija.unios.hr

[3] Department of Pharmaceutical Biotechnology, University of Pécs, Faculty of Pharmacy, Rókus u. 2. H-7624 Pécs, Hungary; banfai.krisztina@pte.hu, bovari.judit@pte.hu, pongracz.e.judit@pte.hu

*corresponding author. E-mail: szechenyi.aleksandar@gytk.pte.hu Phone: +36-70-3814462

Authors declare no conflict of interest.

The Supporting information consist of 12 pages, and contains 6 chapters with 14 figures and 4 tables.

1. Determination of chitosan solubility

The influence of pH and chitosan concentration on chitosan solubility were examined in both malic acid and glutaric acid solutions. In case of examine the influence of pH on solubility, an initial solution was prepared by weight 1.0 m/m% chitosan in 5.0 m/V% dicarboxylic acid solutions, and diluted until the solution turned into a suspension. In case of examine the influence of chitosan concentration on chitosan solubility, an initial solution was prepared by weight 0.25 m/m% chitosan in 2.0 m/V% dicarboxylic acid solution, and added chitosan until the solution turned into a suspension.

The light scattering of solutions were determined visually and with instrumental measurement of scattered light intensity (Zetasizer NanoS, Malvern Panalytical Ltd. UK). The examinations were performed at ambient temperature (25°C). The measurements were carried out in autocorrelation mode, and the following parameters were constant: scattering angle 173°, attenuator 7 and its factor 0.0146, measurement position 0.46 mm.

The result shows, that in case of malic acid the chitosan can be dissolved under 3.22 pH value, which means more than 0.025 m/V% acid concentration. At 2.0 m/V% malic acid concentration (pH=2.27) the chitosan can be dissolved until 2.75 m/m% concentration. In glutaric acid the chitosan can be dissolved under 3.59 pH value (0.05 m/V% acid concentration). At 2.0 m/V% glutaric acid concentration (pH=2.79) the chitosan can be dissolved until 3.0 m/m% concentration.

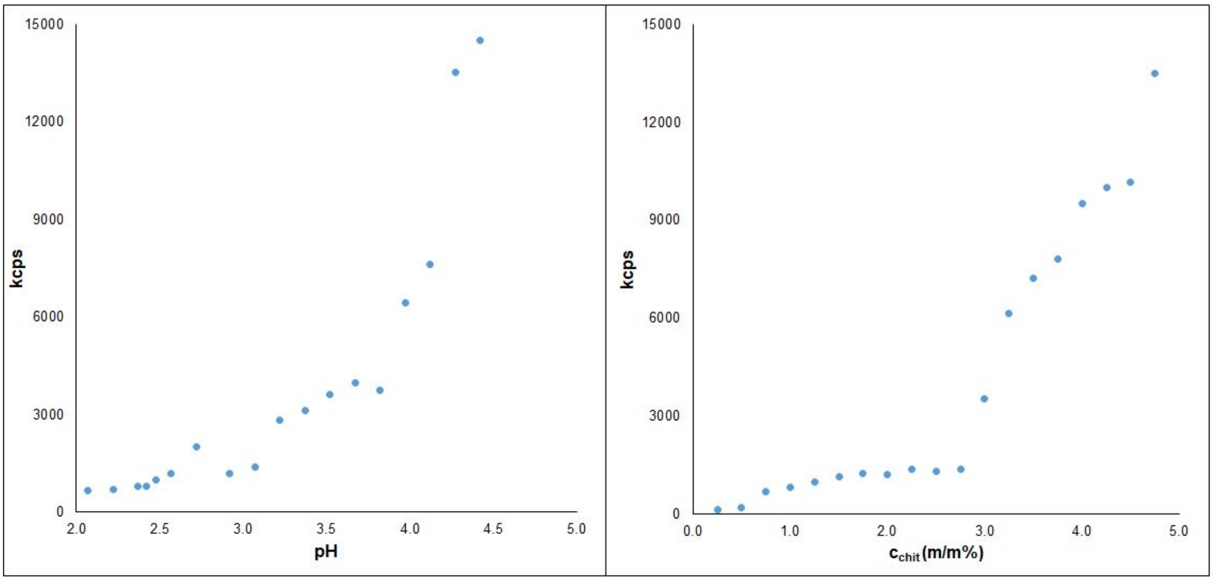

S1. Figure: Determination of chitosan solubility in malic acid with light scattering measurements.

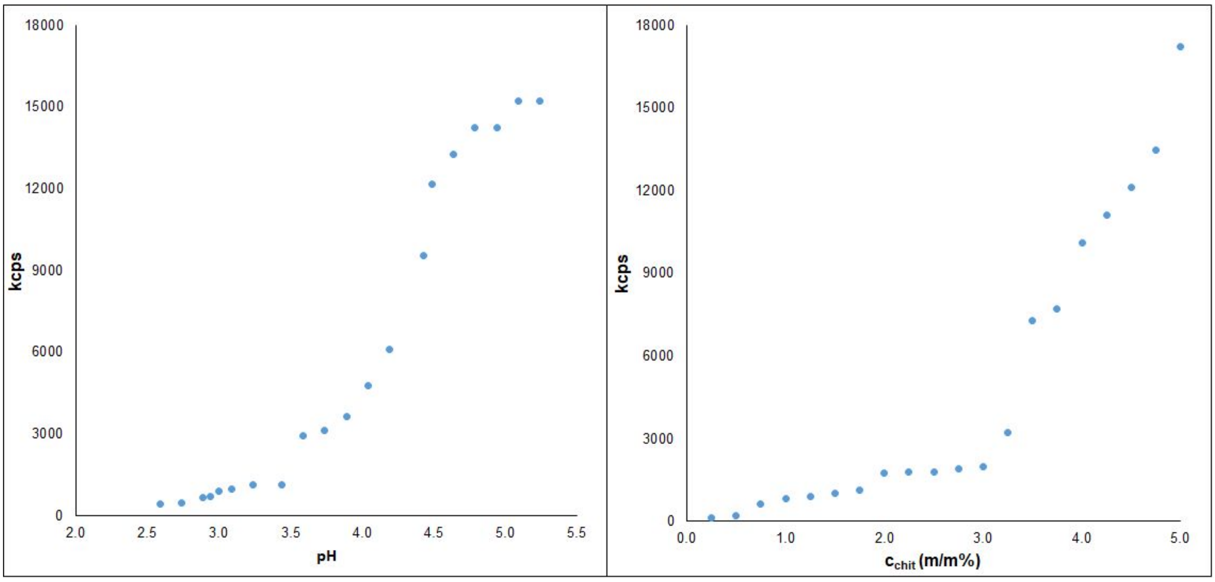

S2. Figure: Determination of chitosan solubility in glutaric acid with light scattering measurements.

2. Results of calculation of cross-linking degree (CR) and cross-linking efficacy (CE)

Sample	n_A (mol/g)	CR (%)	CE (%)	Sample	n_A (mol/g)	CR (%)	CE (%)
Chitosan	$5.25\cdot10^{-3}$	-	-	GA 253	$4.28\cdot10^{-3}$	18.57	3.22
MA 253	$4.23\cdot10^{-3}$	19.52	3.44	GA 258	$4.13\cdot10^{-3}$	21.43	3.72
MA 258	$4.13\cdot10^{-3}$	21.43	3.77	GA 2524	$4.00\cdot10^{-3}$	23.81	4.13
MA 2524	$4.03\cdot10^{-3}$	23.33	4.11	GA 403	$3.70\cdot10^{-3}$	29.52	5.12
MA 403	$4.08\cdot10^{-3}$	22.38	3.94	GA 408	$3.50\cdot10^{-3}$	33.33	5.78
MA 408	$3.88\cdot10^{-3}$	26.19	4.61	GA 4024	$3.43\cdot10^{-3}$	34.76	6.03
MA 4024	$3.83\cdot10^{-3}$	27.14	4.78	GA 503	$2.50\cdot10^{-3}$	52.38	9.08
MA 503	$3.63\cdot10^{-3}$	30.95	9.22	GA 508	$2.38\cdot10^{-3}$	54.76	9.50
MA 508	$3.50\cdot10^{-3}$	33.33	9.64	GA 5024	$2.18\cdot10^{-3}$	58.57	10.16
MA 5024	$3.38\cdot10^{-3}$	35.71	10.31				

S1 Table. Results of determination of number of free amino groups and the cross-linking degree of chitosan and hydrogel samples with conductometric titration. n_A: x mol of free amino group per 1 g chitosan/chitosan hydrogel. CR: cross-linking degree (%). CE: cross-linking efficacy.

3. Determination of flow properties

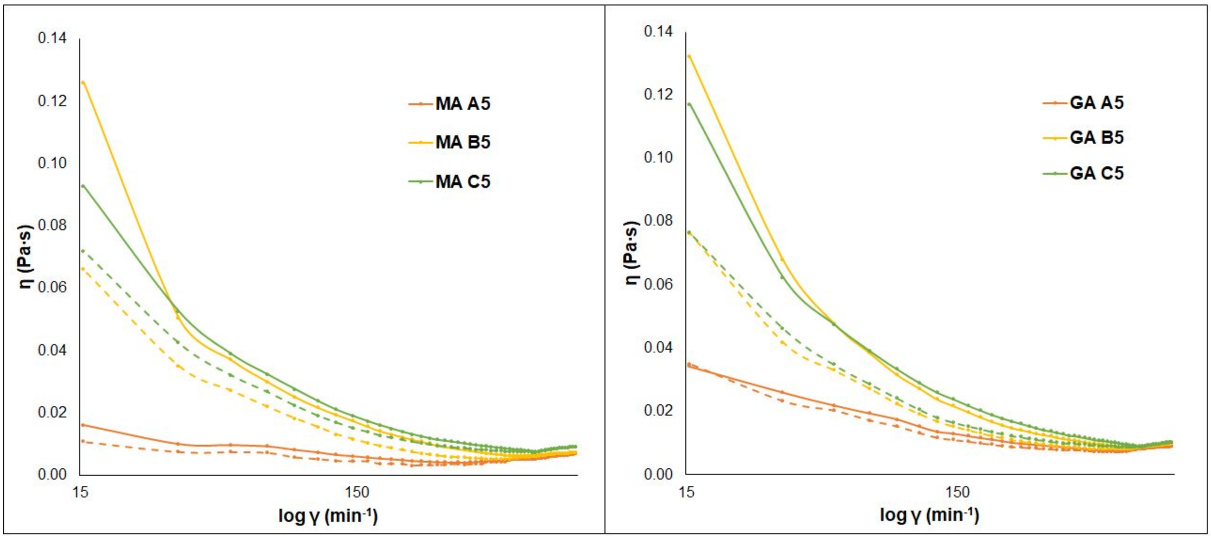

S3 Figure. Viscosity curves of chitosan hydrogels, cross-linked with malic and glutaric acid at different reaction temperatures. The continuous lines remark the upward curves, and the dotted lines remark the downward curves.

Sample	Bingham plasticity parameters			Hysteresis area (Pa/s·ml)	Apparent viscosity (Pa·s)
**MA 298.15**	τ_B (Pa)	η_B (Pa·s)	R^2		
MA 250	0.010	$2.09\cdot10^{-3}$	0.9900	16.744	$4.44\cdot10^{-3}$
MA 251.5	0.018	$2.07\cdot10^{-3}$	0.9822	20.301	$4.55\cdot10^{-3}$
MA 253	0.077	$2.41\cdot10^{-3}$	0.9868	25.606	$4.08\cdot10^{-3}$
MA 25.45	0.143	$3.04\cdot10^{-3}$	0.9715	26.204	$4.57\cdot10^{-3}$
MA 256	0.148	$4.29\cdot10^{-3}$	0.9281	28.000	$5.21\cdot10^{-3}$
MA 258	0.997	$5.01\cdot10^{-3}$	0.9878	34.484	$6.85\cdot10^{-3}$
MA 2524	1.462	$5.51\cdot10^{-3}$	0.9664	54.874	$7.68\cdot10^{-3}$
**MA 313.15 K**	τ_B (Pa)	η_B (Pa·s)	R^2	**Hysteresis area (Pa/s·ml)**	**Apparent viscosity (Pa·s)**
MA 401.5	0.044	$2.77\cdot10^{-3}$	0.9609	23.779	$5.03\cdot10^{-3}$
MA 403	0.053	$3.90\cdot10^{-3}$	0.9695	25.698	$5.99\cdot10^{-3}$
MA 404.5	0.730	$5.43\cdot10^{-3}$	0.9916	35.989	$7.98\cdot10^{-3}$
MA 406	0.747	$6.01\cdot10^{-3}$	0.9325	54.885	$8.54\cdot10^{-3}$
MA 408	1.764	$6.11\cdot10^{-3}$	0.9619	59.790	$9.14\cdot10^{-3}$
MA 4024	2.189	$6.43\cdot10^{-3}$	0.9422	70.833	$9.88\cdot10^{-3}$
**MA 323.15 K**	τ_B (Pa)	η_B (Pa·s)	R^2	**Hysteresis area (Pa/s·ml)**	**Apparent viscosity (Pa·s)**
MA 50.15	0.491	$3.80\cdot10^{-3}$	0.9798	22.456	$6.00\cdot10^{-3}$
MA 503	0.617	$4.06\cdot10^{-3}$	0.9297	34.632	$6.22\cdot10^{-3}$
MA 504.5	1.174	$4.25\cdot10^{-3}$	0.9516	45.619	$6.78\cdot10^{-3}$
MA 506	1.175	$4.92\cdot10^{-3}$	0.9384	73.468	$8.27\cdot10^{-3}$
MA 508	1.480	$5.39\cdot10^{-3}$	0.9876	85.343	$8.72\cdot10^{-3}$
MA 5024	2.338	$3.43\cdot10^{-2}$	0.9468	113.316	$8.67\cdot10^{-3}$

S2 Table. Rheological parameters of chitosan hydrogels cross-linked with malic acid at different temperatures. Chitosan hydrogels show Bingham plastic behaviour. The τB is the yield stress needed to flow (Pa), ηB is Bingham viscosity (Pa·s). R2 provide information about

the fit of Bingham plasticity mathematical model. Hysteresis area values were calculated from the area of hysteresis loop.

Sample	Bingham plasticity parameters			Hysteresis area (Pa/s·ml)	Apparent viscosity (Pa·s)
**GA 298.15 K**	**τ_B (Pa)**	**η_B (Pa·s)**	**R^2**		
GA 250	0.095	$6.14\cdot10^{-3}$	0.9813	14.813	$5.39\cdot10^{-3}$
GA 251.5	0.225	$6.30\cdot10^{-3}$	0.9598	19.463	$7.96\cdot10^{-3}$
GA 253	0.582	$6.94\cdot10^{-3}$	0.9691	26.439	$8.59\cdot10^{-3}$
GA 254.5	0.708	$7.11\cdot10^{-3}$	0.9519	26.820	$8.65\cdot10^{-3}$
GA 256	0.721	$7.13\cdot10^{-3}$	0.9570	34.859	$8.95\cdot10^{-3}$
GA 258	1.234	$7.74\cdot10^{-3}$	0.9788	39.252	$9.34\cdot10^{-3}$
GA 2524	1.521	$8.55\cdot10^{-3}$	0.9820	60.820	$9.38\cdot10^{-3}$
**GA 313.15 K**	**τ_B (Pa)**	**η_B (Pa·s)**	**R^2**	**Hysteresis area (Pa/s·ml)**	**Apparent viscosity (Pa·s)**
GA 401.5	0.502	$6.11\ 10^{-3}$	0.9511	55.463	$8.32\cdot10^{-3}$
GA 403	0.913	$6.11\cdot10^{-3}$	0.9240	70.595	$8.92\cdot10^{-3}$
GA 404.5	1.194	$6.35\cdot10^{-3}$	0.9790	76.055	$8.69\cdot10^{-3}$
GA 406	1.503	$6.55\cdot10^{-3}$	0.9691	77.651	$9.24\cdot10^{-3}$
GA 408	1.628	$6.60\cdot10^{-3}$	0.9632	91.418	$9.83\cdot10^{-3}$
GA 4024	2.002	$7.58\cdot10^{-3}$	0.9693	98.155	$1.03\cdot10^{-2}$
**GA 323.15 K**	**τ_B (Pa)**	**η_B (Pa·s)**	**R^2**	**Hysteresis area (Pa/s·ml)**	**Apparent viscosity (Pa·s)**
GA 501.5	1.156	$6.59\cdot10^{-3}$	0.9815	52.319	$8.69\cdot10^{-3}$
GA 503	1.406	$7.11\cdot10^{-3}$	0.9743	68.531	$9.48\cdot10^{-3}$
GA 504.5	1.468	$7.41\cdot10^{-3}$	0.9571	73.308	$1.01\cdot10^{-2}$
GA 506	1.561	$7.96\cdot10^{-3}$	0.9718	84.826	$1.04\cdot10^{-2}$
GA 508	1.874	$8.05\cdot10^{-3}$	0.9532	89.969	$1.07\cdot10^{-2}$
GA 5024	2.390	$9.12\cdot10^{-3}$	0.9693	117.505	$1.12\cdot10^{-2}$

S3 Table. Rheological parameters of chitosan hydrogels cross-linked with glutaric acid at different temperatures. Chitosan hydrogels show Bingham plastic behaviour. The τ_B is the yield stress needed to flow (Pa), η_B is Bingham viscosity (Pa·s). R^2 provide information about the fit of Bingham plasticity mathematical model. Hysteresis area values were calculated from the area of hysteresis loop.

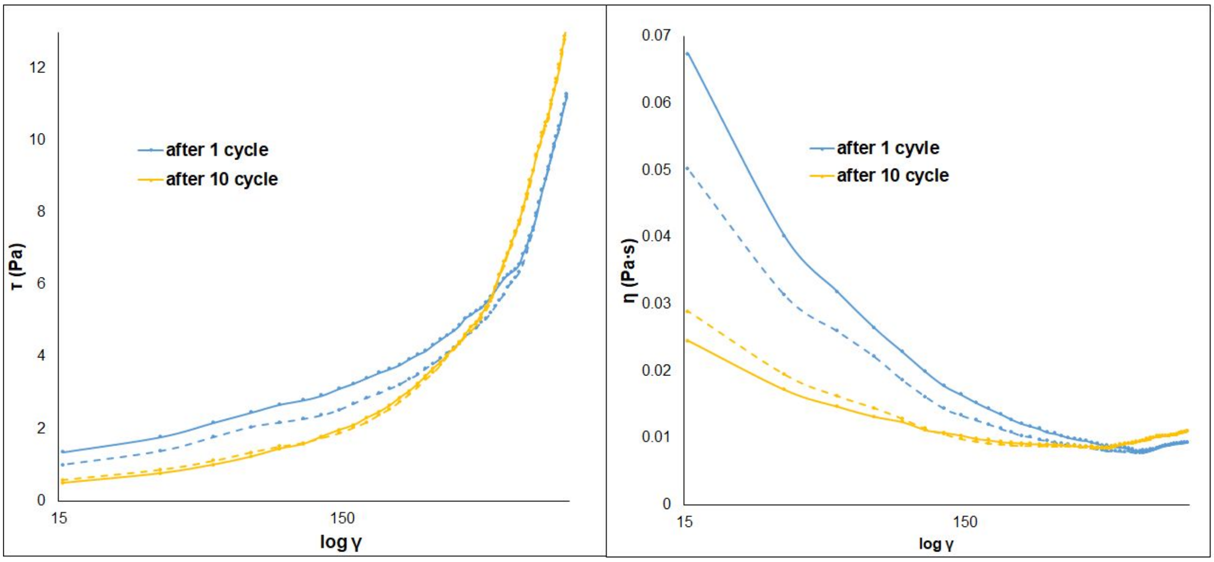

S4 Figure. Flow (A) and viscosity (B) curves of GA 2524 hydrogel sample after 1st and 10th measurement cycle. The continuous lines remark the upward curves, and the dotted lines remark the downward curves.

4. DSC measurements of hydrogels

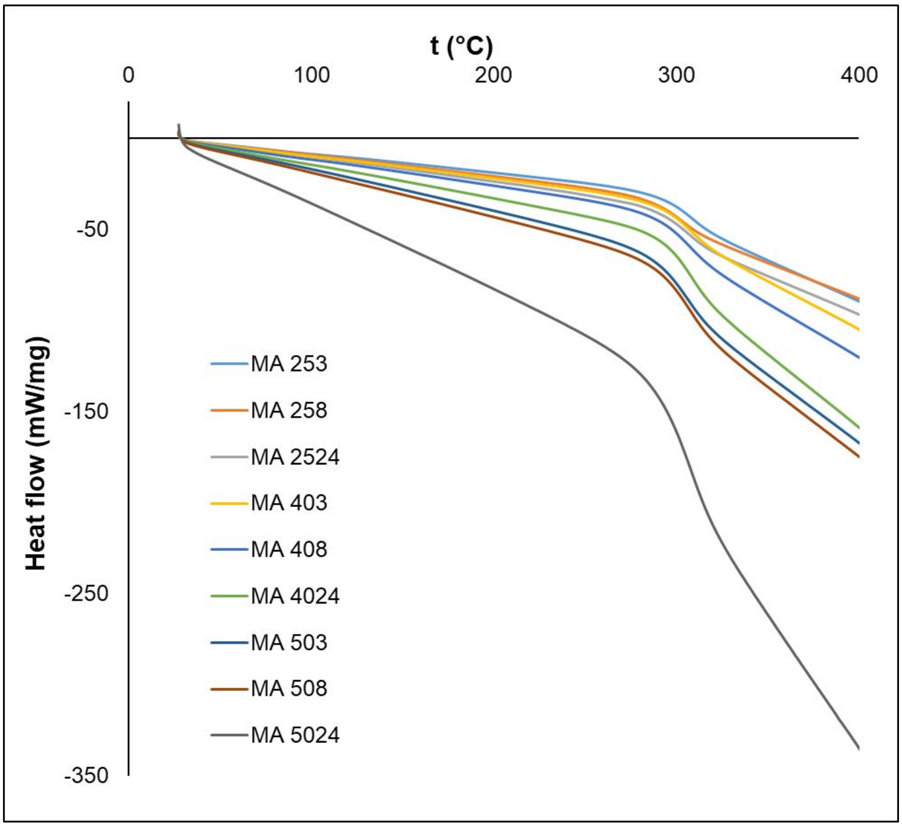

S5. Figure: DSC curve of hydrogels cross-linked with malic acid.

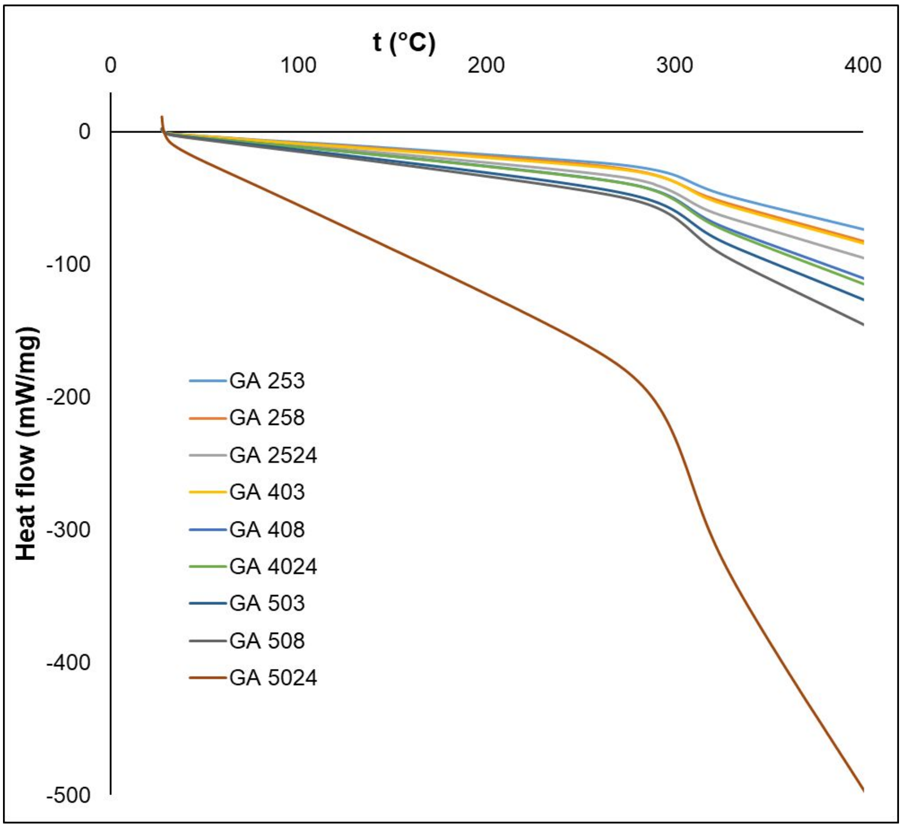

S6. Figure: DSC curve of hydrogels cross-linked with glutaric acid.

5. Characterization of API adsorption of hydrogels

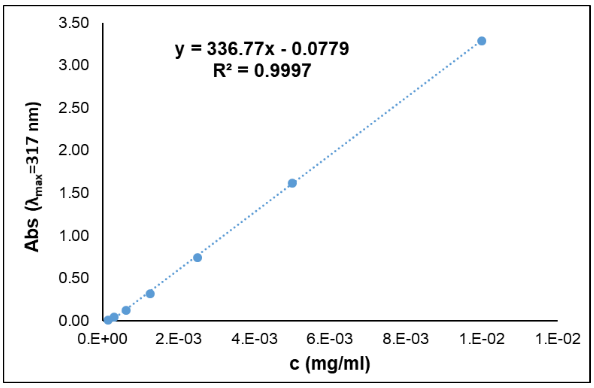

S7 Figure: Calibration curve of baicalin.

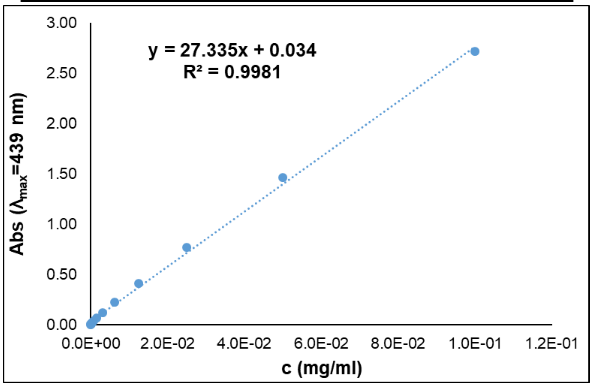

S8 Figure: Calibration curve of emodin.

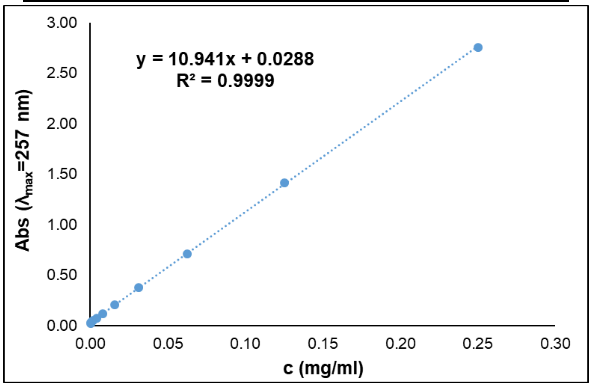

S9 Figure: Calibration curve of glycyrrhizic acid.

API	Linearity	LOD (mg/ml)	LOQ (mg/ml)
baicalin	0.997	0.000106	0.000321
emodin	0.9981	0.00236	0.00715
glycyrrhizic acid	0.9999	0.00126	0.00367

S4 Table: General analytical parameters of APIs.

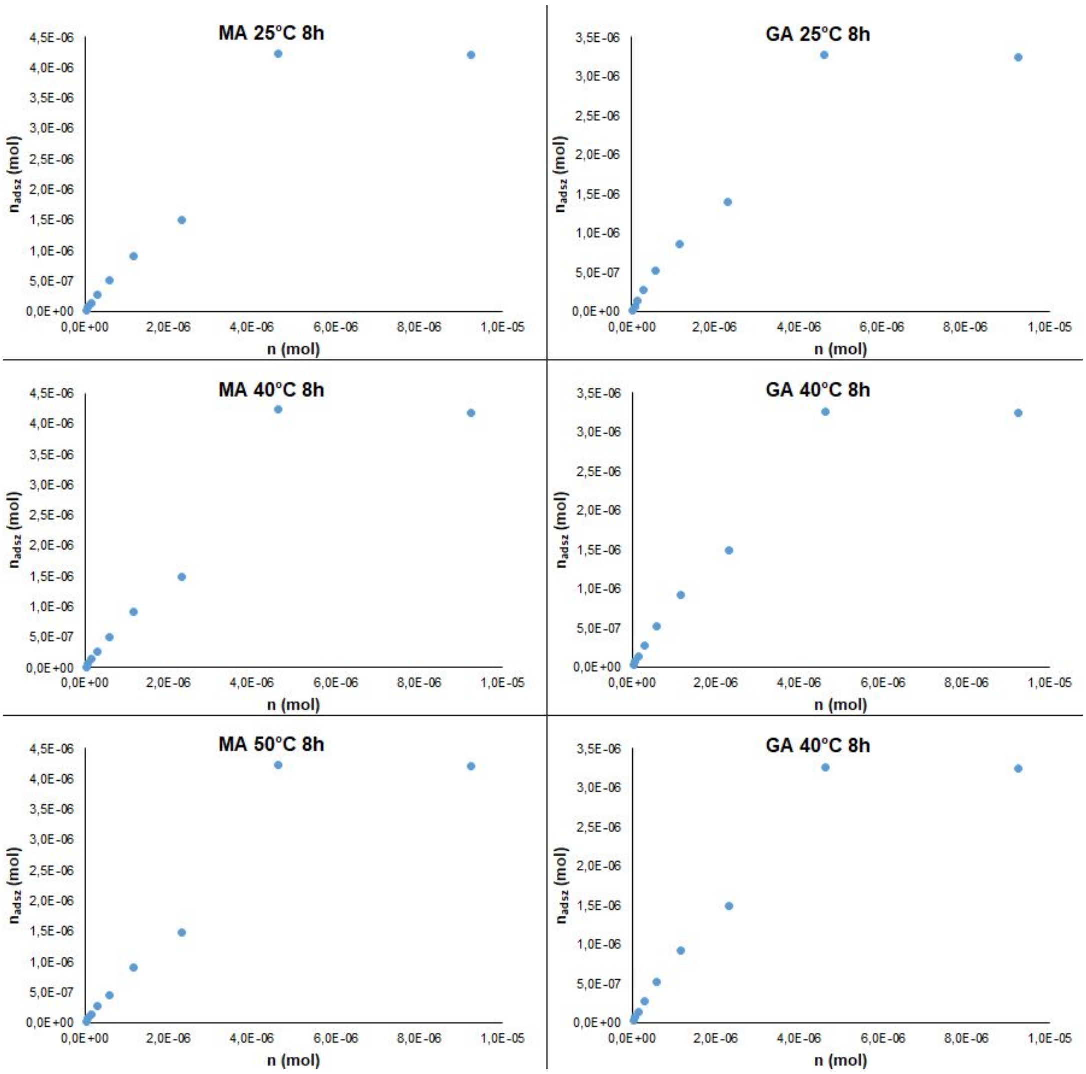

S10. Figure: Adsorption isotherm of chitosan hydrogels with emodin. MA: hydrogels cross-linked with malic acid. GA: hydrogels cross-linked with glutaric acid.

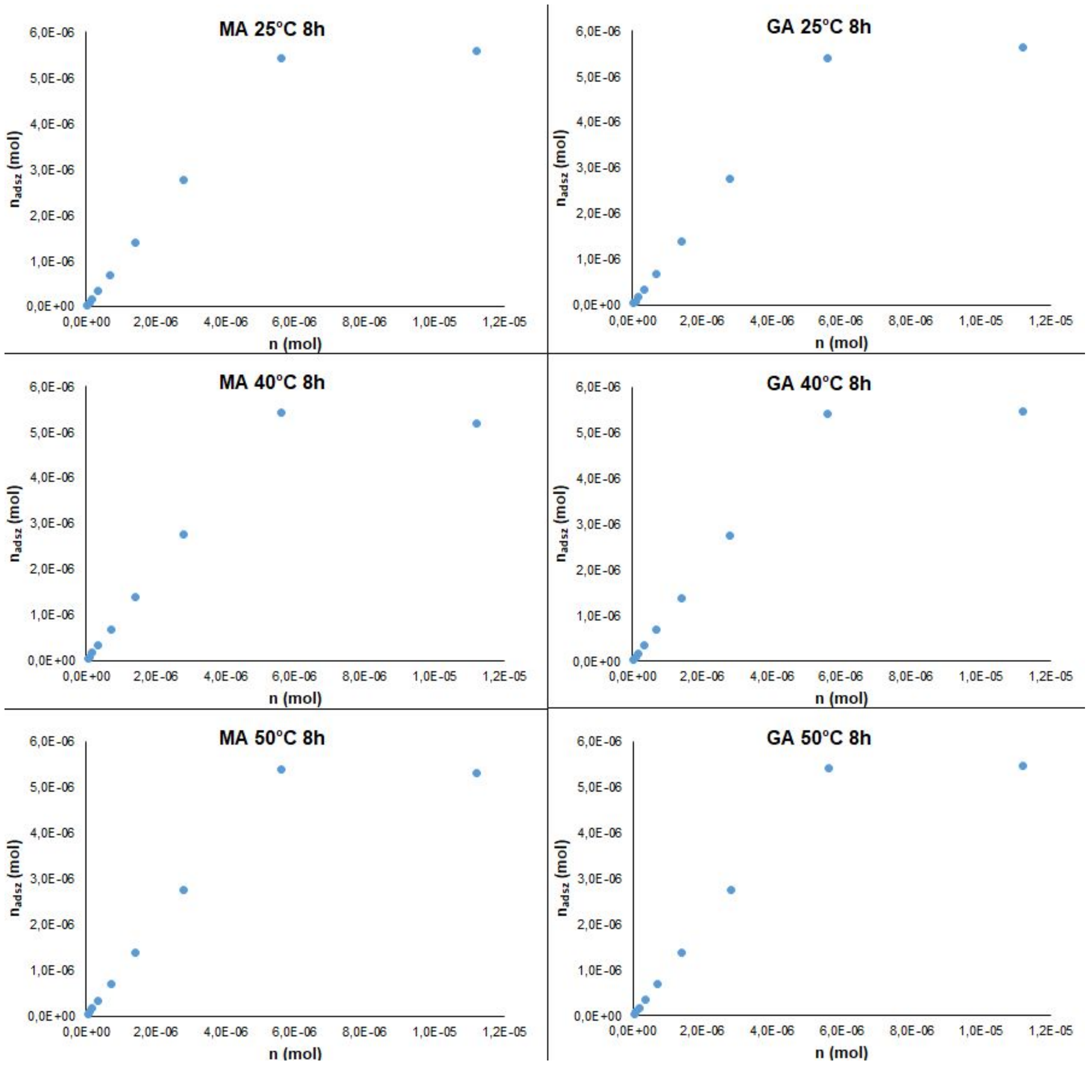

S11. Figure: Adsorption isotherm of chitosan hydrogels with baicalin. MA: hydrogels cross-linked with malic acid. GA: hydrogels cross-linked with glutaric acid.

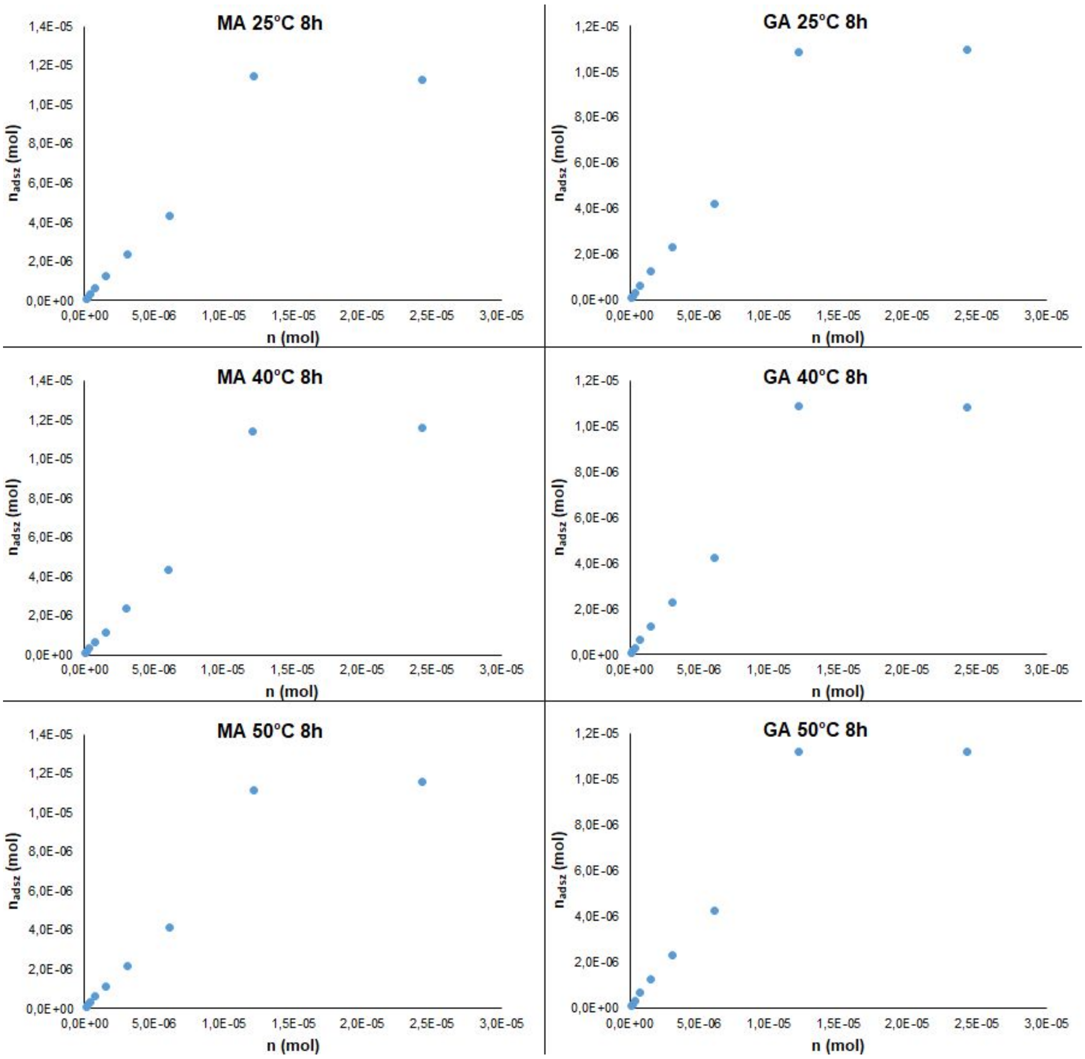

S12. Figure: Adsorption isotherm of chitosan hydrogels with glycyrrhizic acid. MA: hydrogels cross-linked with malic acid. GA: hydrogels cross-linked with glutaric acid.

6. Results of cytotoxicity measurements of hydrogels

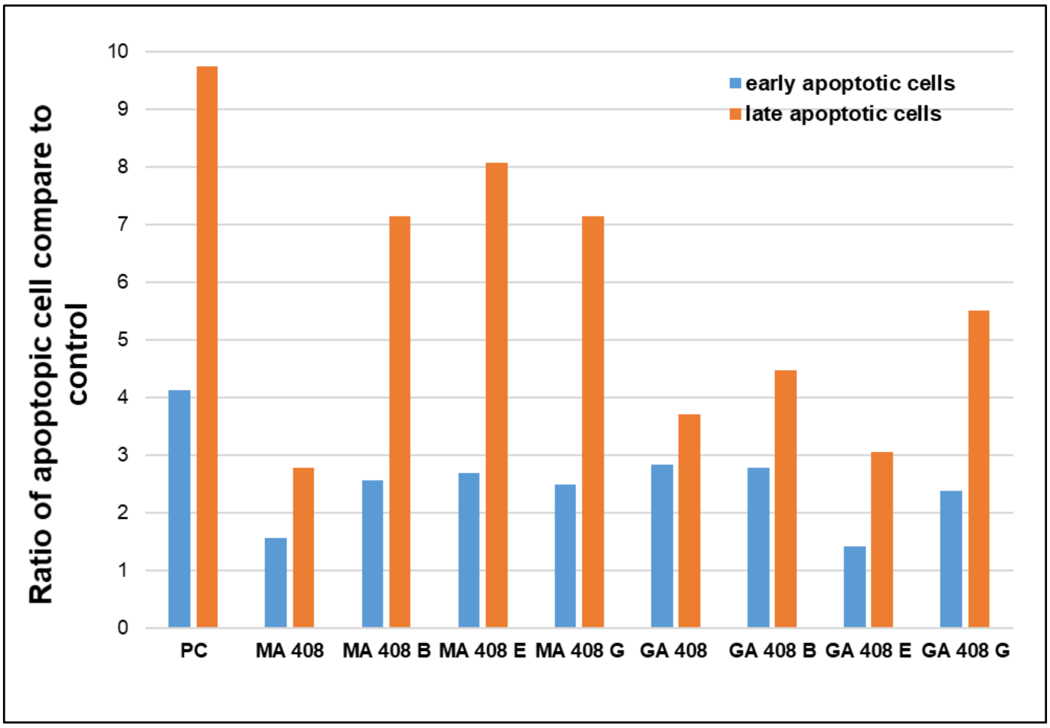

S13. Figure: Results of cytotoxicity measurements of HEK 293 cell lines. The ratio of apoptotic cells was calculated to the untreated sample. The concentration of hydrogels were 0.1 mg/ml. PC: positive control (3 V/V% DMOS solution). B: baicalin. E: emodin. G: glycyrrhizic acid.

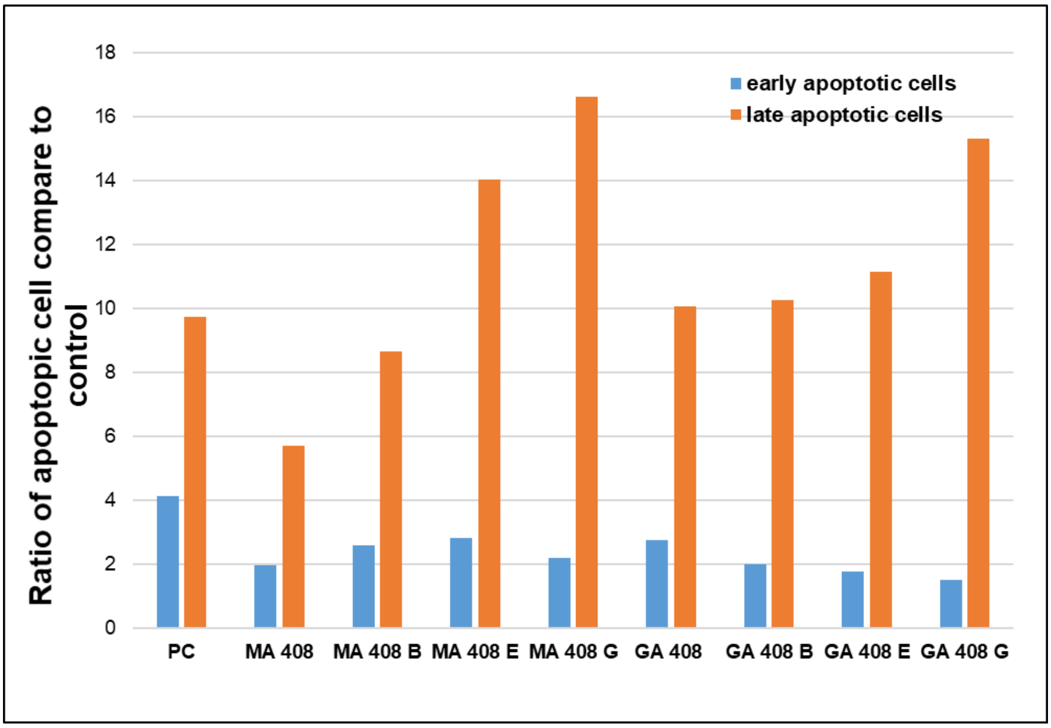

S14. Figure: Results of cytotoxicity measurements of HEK 293 cell lines. The ratio of apoptotic cells was calculated to the untreated sample. The concentration of hydrogels were 10 mg/ml. PC: positive control (3 V/V% DMOS solution). B: baicalin. E: emodin. G: glycyrrhizic acid.
